# Advancing the allostatic load model in military training research: from theory to application

**DOI:** 10.3389/fphys.2025.1638451

**Published:** 2025-09-26

**Authors:** Evan D. Feigel, Kristen J. Koltun, Mita Lovalekar, Karl E. Friedl, Brian J. Martin, Bradley C. Nindl

**Affiliations:** ^1^ Neuromuscular Research Laboratory/Warrior Human Performance Research Center, University of Pittsburgh, Pittsburgh, PA, United States; ^2^ US Army Research Institute of Environmental Medicine, Natick, MA, United States

**Keywords:** stress, regulation, allostasis, wearable, performance, musculo skeletal disorder, psychological distress

## Abstract

Research physiologists use theoretical models to test new empirical relationships between physiological variables and psycho-physiological outcomes and compare observed outcomes with theoretical predictions to support or refute models. Models, while valuable, often focus on a limited perspective as part of a larger reality. In understanding Warfighter health, a more holistic perspective within a model is needed since this population is exposed to a high degree of physical, cognitive, and emotional demands/loads during training throughout a career. Focusing on the physical performance aspects of occupational exposures is important; however, this neglects imperative interrelationships between the psychological and musculoskeletal domains of health, which must be quantified for early in-field prevention of injury, underperformance, or psychological harm. Chronic duration of the physiological stress response may disrupt adaptive mechanisms and result in allostatic load, characterized as a maladaptive biological process by which physiological stability (‘allostasis’) fails owing to repeated and chronic stress exposure, which can negatively affect physical and cognitive function. It may also increase vulnerability to atypical reductions in occupational physical performance and psychological and musculoskeletal health. The purpose of this review was to (i) summarize empirical research of atypical, negative consequences of military training on physical performance and psychological and musculoskeletal health (ii); reconsider the underlying biological process rendering maladaptive outcomes observed during training by leveraging a ‘stress perspective’ wherein military training-related stressors perturb stress systems and lead to allostatic load, which may serve as a mechanism by which maladaptation occurs; (iii) summarize the impact of allostatic load quantified by the Allostatic Load Index (ALI) on physical performance, psychological wellbeing, and musculoskeletal health; and (iv) propose the use of valid and reliable commercially-available wearable devices as tools to measure allostatic load by collecting longitudinal cardiometabolic and neurobehavioral (sleep) data during training and determining verifiable signals associated with ALI and maladaptive outcomes. Allostatic load is an evolving model that may be suited to understand the long-term health effects of military training-related stress. There is opportunity to improve our understanding of measurement tools involving wearables to establishing the relationship between allostatic load and long-term health outcomes in military personnel.

## 1 Introduction

Warfighters are characterized as military service members directly engaged in combat-related roles and/or military operations (Nindl et al., 2017). In the United States (US), Warfighter readiness, lethality, and resilience remain a continued priority in the US Armed Forces as evidenced by the enacted “Military Readiness and Injury Prevention Act of 2019” (S.1860) (Moran and Smith, 2019), the Holistic Health and Fitness (H2F) Initiative of 2020 (U.S. Army Center for Initial Military Training, 2023), the Brandon Act of 2023 to advance mental health supportive programs (Military Health System, 2023), and the Defense Health Agency’s 6-year advancement plan (2021–2027) to leverage fitness wearable devices for measuring and promoting readiness (Cisneros, 2023). Together, such initiatives may define the optimal Warfighter as one who is healthy enough to operate on short notice with or without appropriate recovery, resilient enough to overcome environmental, internal (biogenic, physiological), and external (mechanical, social) stressors while maintaining occupational role performance, and robust against the occurrence of musculoskeletal injury (MSKI) (Moran and Smith, 2019; U.S. Army Center for Initial Military Training, 2023; Military Health System, 2023; Cisneros, 2023).

Pertinent to the operational success of the Warfighter is the execution and completion of military training courses to learn and excel in physical, academic, and tactical skills for deployment, accrue individual military rank, and extend one’s military career (Drain et al., 2015; Caspar et al., 2020; Bartlett et al., 2015). Warfighters spend considerable amounts of time each year enrolled in such courses, ranging between eight and 12 weeks for the majority of courses (Bulmer et al., 2022a; Alemany et al., 2008; Henning et al., 2014; Harman et al., 2008), with specific curricula ranging four to 6-weeks in duration (Edgar et al., 2021). Although their curriculum aims to enhance physical and psychological readiness for deployment (Flanagan et al., 2012), empirical research over 3 decades pinpoints the atypical negative effects of training, including MSKIs (Jones et al., 2010), worsened physical performance (Burley et al., 2018) and lower psychological wellbeing (Bulmer et al., 2022a). Together, these effects contribute to attrition owing to deterred medical or psychological health (Tait et al., 2022; Forse et al., 2024) and financial burden on healthcare systems (Dijksma et al., 2020).

Prevention initiatives within the US Armed Forces that aimed to tackle such negative effects observed during training have implemented deductive approaches wherein accrued findings from national databases of military health records result in advanced programs to address prevention, early identification, and management of negative effects (Cooper et al., 2024). Such programs also aimed to rescue financial security in the military domain (Cooper et al., 2024). Previous research demonstrated beneficial results of programs targeting musculoskeletal (Wardle and Greeves, 2017), physical fitness (Burley et al., 2020), and psychological health (Adler et al., 2015) when employed independently. However, although holistic health programs, such as the H2F Initiative, which aims to implement preventive care for soldiers due to increasing sleep and mental health concerns, and MSKI rates (Culley and DaLomba, 2025), have been a mainstay within US brigades since 2020, few interventions have assessed its effectiveness on musculoskeletal, physical fitness, and psychological health (Cooper et al., 2024). A 2025 study observed an increase in awareness of H2F in soldiers without assessing its effectiveness on targeted outcomes (Culley and DaLomba, 2025). Hence, as opposed to employing a deductive mechanism that relies on a reactive technique that leads to actionable programs, implementing an inductive mechanism by reconsidering the underlying biological processes that may render negative outcomes, such as allostatic load (McEwen, 1993), may improve our understanding of their development and advance preventive means.

**FIGURE 1:**
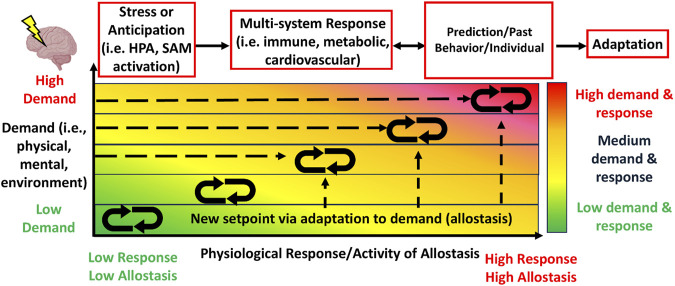
The allostasis model illustrates the constant dynamism of physiological activity of stress-related systems, including activity from the hypothalamic-pituitary-adrenal (HPA) and sympathetic-adrenal-medullary (SAM) systems, that serve to appropriately respond to physical, mental or environmental stressors in order to adapt to the physical, mental or environmental demands in a one-to-one ratio. Stress or anticipation of a stressor activates a physiological response to which leads to multi-system (i.e., immune, cardiovascular) responses. The physiological response is mediated and influenced by individual prediction of the stressor, one’s past experience or behavior, and individual factors (i.e., age), resulting in adaptation. With each increase in demand requires a met response of the body, which always stays in dynamic flux (McEwen, 1993).

**FIGURE 2:**
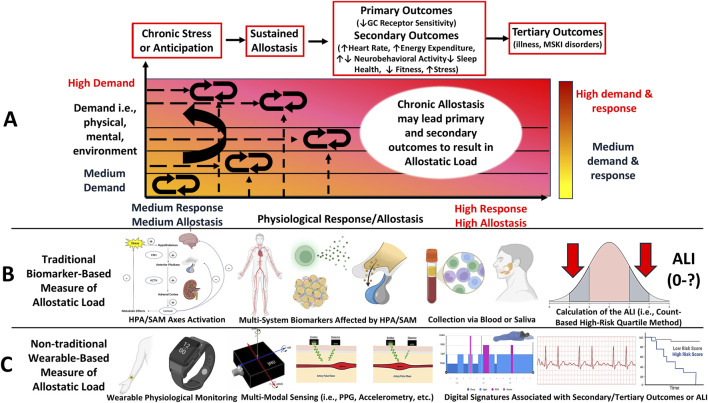
Letter **(A)**: The Allostatic Load model depicts how chronic or repeated stress exposure disrupts allostasis, leading to physiological dysregulation, characterized as an inability to adapt to the demands, which leads to negative health outcomes. Key elements of this model include *primary outcomes*, including degradation of protective adaptive mechanisms from the stress response driven by negative feedback loops, *secondary outcomes*, including the downstream physiological effects of primary outcomes, such as elevated cardiometabolic and altered neurobehavioral activity, and behavioral responses, such as worsened fitness and psychological well-being. *Tertiary outcomes* are characterized as the maladaptive consequences of *secondary outcomes*, including musculoskeletal and psychological disorders and illnesses. Allostatic load is deemed experienced when chronic stress is met with measurable secondary or tertiary outcomes. Unlike allostasis, which shows a one-to-one response to demand ratio, the increased demands (y-axis) eventually leads to *primary outcomes*, which shifts the response (x-axis) to the left under heightened demands. This leads to maladaptive outcomes, thus resulting in Allostatic Load. Letter **(B)**: This model can be measured using traditional, biomarker-based methods. Biomarker-based methods require multi-system biomarker data from stress systems collected by a medium and summarized into quartiles. The quartiles can be used as cut-off thresholds to compute the Allostatic Load Index, that is, the number of biomarkers falling within at-risk quartiles commonly associated with stress pathophysiology. A higher index value is an indicator of greater allostatic load being experienced. Letter **(C)**: This model may also be measured using non-traditional, wearable-based methods, which may be particularly suited for military training environments. Signal features, including photoplethysmography (PPG) and triaxial accelerometry, can be used to measure physiological responses to training that may be associated with secondary and tertiary outcomes. Such associations may reveal a digital phenotype of allostatic load (Figure 4), however, this remains theoretical.

Allostatic load is a theoretical biological framework that outlines a maladaptive biological process wherein physiological stability, known as allostasis (Sterling, 1988) (Figure 1), characterized as the constant dynamism of physiological activity of biological systems that appropriately responds to stressors to maintain homeostasis, fails owing to dysregulated primary mediator activity from chronic stress exposure and reflects the cumulative physiological burden (McEwen BS., 1998). Allostatic load is a stress regulation model that describes how the ‘cost’ of adaptation to chronic stress exposure may result in multi-system dysregulation (Sterling, 1988) (Figure 2A). This model is reflected by the presence of *primary outcomes* or effects, characterized as the degradation of protective mechanisms that mediate physiological stress responses (i.e., desensitization of glucocorticoid receptors) and *secondary outcomes* or effects (Guidi et al., 2020)*,* characterized as consequential physiological responses from primary outcomes reflected by heightened cardiometabolic and altered neurobehavioral (i.e., sleep) health (McEwen, 2006; Logan and Barksdale, 2008; Feigel et al., 2024a), as well as adverse behavioral responses, such as worsened physical performance (Germano et al., 2023), sleep quality (Christensen et al., 2022), self-appraised resilience (Felix et al., 2023), and perceived stress appraisal (McEwen, 2007). *Tertiary outcomes* emerge from secondary outcomes*,* such as pain syndromes, musculoskeletal disorders, and illnesses (Feigel et al., 2024a; Beckie et al., 2016; Parker et al., 2022) (Figure 2A). Taken together, this model highlights the influence of the environment, individual variation, and brain-body interactions to serve as a process by which chronic stress may result in secondary (i.e., poor fitness) and tertiary outcomes (i.e., MSKI) during training (McEwen BS., 1998; Feigel et al., 2024a).

Allostatic load is measured by its traditional operationalization, the Allostatic Load Index (ALI) (Figure 2B), which comprises a count-based composite score representing the number of biomarkers affected by chronic psycho-physiological stress across neuroendocrine, autonomic, and immune systems (Juster et al., 2010; Seeman et al., 1997). The ALI score is determined by the number of biomarkers collected with higher scores indicating higher allostatic load (McLoughlin et al., 2020; Carbone et al., 2022) (Figure 2B). The use of the ALI to quantify allostatic load has grown considerably (Juster et al., 2010; Seeman et al., 1997; Geronimus et al., 2006). This has been shown in epidemiological studies (Carbone, 2021; Andrzejak et al., 2023; Bruun-Rasmussen et al., 2024), which have associated ALI with several negative health outcomes, including subclinical risk factors for cardiovascular disease (Logan and Barksdale, 2008), heightened morbidity and mortality rates (Parker et al., 2022; Bruun-Rasmussen et al., 2024), accelerated mechanisms of aging (Seeman et al., 1997), psychological disorders (Beckie et al., 2016; Berger et al., 2019; Berger et al., 2018), physical locomotor dysfunction and musculoskeletal disorders (Germano et al., 2023), reduced muscular strength and postural balance (Germano et al., 2023; Hansen et al., 2016), and worsened psychological wellbeing, such as symptoms of anxiety (Gou et al., 2025; D’Alessio et al., 2020) and depression (Gou et al., 2025) and lower resilience (Felix et al., 2023), sleep quality (Christensen et al., 2022), and perceived stress (Guidi et al., 2020; Beckie et al., 2016; Juster et al., 2010; Geronimus et al., 2006).

Recent work from our group observed an increased ALI score following a 10-week military training course in both sexes (males: +2 ALI (5 out of 8); females: +1 ALI (4 out of 8)), and observed an association with worsened physical performance to support this framework as a useful model outlining a biological process associated with maladaptive training-related outcomes (Feigel et al., 2025a). Since the origination of the ALI in 1997 (Seeman et al., 1997), the number of biomarkers and algorithms used for the ALI has grown (McLoughlin et al., 2020; Carbone et al., 2022). However, this method has limitations for longitudinal study designs (Feigel et al., 2025a; Magtibay and Umapathy, 2023), including obtaining several biomarkers multiple times over a study duration, which may render the ALI a challenging method for military training environments (Feigel et al., 2025a). Therefore, as an alternative approach, recent studies have begun to employ non-traditional, commercial wearable-based methods for its assessment (Magtibay and Umapathy, 2023; Corrigan et al., 2021; Corrigan et al., 2023) (Figure 2C). Wearable-based methods may be a promising approach for allostatic load assessment owing to devices’ noninvasive wear and their ability to capture high-resolution physiological time-series data from multiple sensors (Figure 2C). Importantly, such tools may reveal digital signatures and serve as a proxy measures of the ALI (Magtibay and Umapathy, 2023). However, empirical research on this phenomenon remains unexplored. Moreover, although the ALI has been examined in military populations (Feigel et al., 2025a), limitations for wearable-based methods, such as their methodological variability and data quality, should be considered for its feasibility for employment (Feigel et al., 2024a; Magtibay and Umapathy, 2023).

The purpose of this review is to (i) summarize the empirical research of atypical, negative consequences of military training on physical performance and psychological and musculoskeletal health (ii); reconsider the underlying biological process rendering maladaptive outcomes commonly observed during training by leveraging a ‘stress perspective’ where military training-related stressors, such as energy restriction, physical overtraining, cognitive distress, and sleep deprivation, perturb stress systems and lead to allostatic load, which may serve as a mechanism of training-related maladaptation; (iii) summarize the empirical research of allostatic load quantified by the ALI on physical performance, psychological wellbeing, and musculoskeletal health; and (iv) propose the use of valid and reliable commercial wearable devices as tools to measure allostatic load by collecting longitudinal cardiometabolic and neurobehavioral data during training and determining verifiable signals associated with ALI and maladaptive outcomes. Such findings may reveal a ‘digital phenotype’ of allostatic load for in-field detection and introduce a field-expedient method to identify personnel at-risk for maladaptive outcomes that occur during military training courses.

## 2 Musculoskeletal, physical performance and psychological maladaptation to military training

In the US Armed Forces, across all branches (Army, Navy, Air Force, and Marines), ∼5% of all hospitalizations have been attributed to MSKIs, with ∼90% classified as ‘non-combat’ MSKIs (Hauret et al., 2010). ‘Non-combat’ MSKIs are characterized as those MSKIs that occur not from ballistic weaponry, improvised explosive devices (IED), parachuting, or vehicular accidents (i.e., helicopter crashes), and remain the leading cause of outpatient medical care in the US Army of active duty personnel, with two million encounters each year (Molloy et al., 2020a). ‘Non-combat’ designated MSKIs of the upper and lower body regions account for 80% of all observed MSKIs (Molloy et al., 2020a; Molloy et al., 2020b), 50%–75% of those sustained in the lower body, including lumbar/sacral spine, pelvis, and lower extremity, MSKIs (Jones et al., 2010; Hauschild et al., 2018; Lovalekar et al., 2018). Notably, the hip, ankle, and foot account for 14%, 12%, and 12% of all lower body MSKIs, respectively (Hauschild et al., 2018). Importantly, ∼60% of limited duty days and 65% of non-deployable Warfighters have been attributed to non-combat MSKIs (Flanagan et al., 2012; Jones et al., 2010). The majority (∼85%) (Hauschild et al., 2018) of MSKIs sustained are categorized as ‘mechanical’ owing to external shear forces induced upon the musculoskeletal system, with ∼75% categorized as ‘overuse’ owing to the cumulative microtrauma (i.e., repetitive stress) and 10% as non-contact acute trauma (non-ballistic) MSKIs (Jones et al., 2010; Hauschild et al., 2018; Jensen et al., 2019).

Overuse MSKIs observed during military training range in type from bone stress fractures (Molloy et al., 2020a; Molloy et al., 2020b; Koltun et al., 2022), inflammation and pain (Hauret et al., 2010), sprains and strains (Lovalekar et al., 2018; Lovalekar et al., 2023; Lovalekar et al., 2021), and even soft tissue degenerative diseases, such as osteoarthritis (Showery et al., 2016; Knapik et al., 2018). Lovalekar et al. (2023) (Lovalekar et al., 2023) observed a cumulative MSKI incidence of 39.7% in women and 23.1% in men among 736 US Marine candidates (n = 131 women), with ∼65% categorized as overuse MSKIs (Lovalekar et al., 2023). Overuse MSKIs have been suggested to occur from the rigorous and physically demanding training involved (Allison et al., 2017). Additionally, overuse MSKI is an important cause of attrition, disability, and loss of military readiness (Songer and LaPorte, 2000) and high financial cost (Lovalekar et al., 2018). A 2018 study assessing the cost of such MSKIs among Air Force Special Tactics Operators showed that the total lifetime cost sustained by them during only a 1-year period was US $1.2 million (Lovalekar et al., 2018). Notably, a 2000 retrospective cohort study describing the MSKI occurrence during a 6-week United States Marine Corps (USMC) Officer Candidates School (OCS) course among 480 candidates (n = 30 women) observed a cumulative incidence (one or more MSKIs) of 60.7% (women: 80.0%; men: 59.5%) with overuse MSKIs making up 65.2% of all encounters in men, and 70.3% of encounters in women. Together, overuse MSKIs were responsible for 0.62 and 1.67 lost training days per man and woman, respectively (Piantanida et al., 2000). However, a 2023 retrospective cohort study of OCS candidates undergoing this course reported a lower cumulative injury incidence of 39.7% in women (n = 52) and 23.1% (n = 140) in men. Although these results show improvement in overuse MSKI rates, further analysis reveals that such MSKIs remained the predominant MSKI type in both sexes (women: 66.2%; men: 65.4%), suggesting that they remain a medical challenge as they were 2 decades ago (Lovalekar et al., 2023).

Although overuse MSKIs remain a prevalent challenge in contemporary training courses, recent evidence also observes atypical reductions in physical performance following training in both sexes (Brock and Legg, 1997; Booth et al., 2006; Tanskanen et al., 2011; Givens et al., 2023a). Physical fitness is emphasized as a critical element for advancement early in military servicemember careers (Agostinelli et al., 2022) that predict the success of military job-task roles (Bartlett et al., 2015; East et al., 2017). This is due to the physically demanding and commonly recurring gender-neutral tasks soldiers are required to perform in ground close combat roles (Nindl et al., 2015; Szivak and Kraemer, 2015). Additionally, the uplift of bans for women preventing enrollment in ground close combat in nations of the North Atlantic Treaty Organization (NATO) has positioned research on the physiological effects of physical training on male and female servicemembers to the forefront (Fitriani and Matthews, 2016; Sterczala et al., 2023; Feigel et al., 2024b). Recent research has shown that lowered fitness during training may pose problems for role performance (Alemany et al., 2008; Allison et al., 2017), and risk of detraining and lethality during deployment (Pihlainen et al., 2023). Decreases of 15%–20% in aerobic capacity and 10% in maximal muscular strength have been observed in both sexes following training (Häkkinen et al., 1985). Burley et al. found that 15% and 14% of recruits showed a significant decline (≥5%) in maximal muscular strength assessed by a 1 repetition-maximum box lift and local muscular endurance assessed by a maximum number of pushups achieved in 2-min (Burley et al., 2018). Further analysis from this same investigation observed that a total of 7% and 4% of the sample reduced in estimated VO2_peak_ and 3.2 km load carriage performance (≥5%), respectively (Burley et al., 2018). Similarly, Givens et al. observed no significant improvement in upper body muscular endurance (count: 6 ± 1 vs. 6 ± 1, p > 0.05) or aerobic capacity (25:14 ± 0:15 vs. 24:51 ± 0:15, p > 0.05) following a 10-week course in female US Marines (Givens et al., 2023a). Although these results also depend upon training length and specificity (Coge et al., 2024), fatigue accumulation (Heilbronn et al., 2023), and motivation (Myllylä et al., 2023), such results demonstrate a divergent performance response to training in cohorts undergoing identical physical training curricula.

In addition to observed atypical reductions in physical performance, previous investigations have observed individuals (10%–25%) (Robinson et al., 2009) demonstrating lower levels of psychological wellbeing during training that can contribute to volitional attrition rates up to 25.8% (Forse et al., 2024). Lower psychological wellbeing during military training (Forse et al., 2024; Beckie et al., 2016; Bulmer et al., 2022b; McFadden et al., 2024a) may be characterized as the independent or combined experience of one or more of the following: (i) lower self-appraised psychological resilience (Forse et al., 2024; Nindl et al., 2018; McFadden et al., 2024b), a dampened degree to which an individual copes in stressful situations or during times of adversity (Connor and Davidson, 2003), (ii) higher perceived stress appraisal, a heightened degree of self-reported perception of situations being particularly stressful (Cohen et al., 1983), and (iii) worsened subjective sleep difficulty, a higher degree to which individuals feel that they struggle to attain a sufficient night of sleep (Bender et al., 2018; Kargl et al., 2024). Recent investigations have observed a link between the aforementioned symptomatology and risk of readiness for deployment (Paxton et al., 2024), and incidence of psychological disorders (Beckie et al., 2016), including post-traumatic stress disorder (PTSD) (Abouzeid et al., 2012). Active duty personnel are likely to worsen symptoms concerning suicidal ideation (OR = 1.90, 95% CI = 1.20–2.90) following training compared to reservists (Robinson et al., 2009). A retrospective cohort study that assessed a battery of psycho-physiological characteristics of 1006 OCS candidates (79.5% male) observed that lower self-appraised resilience was amongst the main predictors of attrition (Forse et al., 2024). Although perceived stress has been shown to improve cognitive focus and motivation to foster effective learning (Ross et al., 2024), chronic, heightened levels of stress can dampen cognitive performance (Ross et al., 2024) and risk discharge (Taylor et al., 2009). Taylor et al. (2009) (Taylor et al., 2009) observed that perceived stress was directly associated with acute stress biomarkers, whereas active coping ability was not, suggesting that perceived stress plays a fundamental role in the physiological stress response (Taylor et al., 2009). Among 202,339 active duty enlisted US Air Force trainees, 50% reported sleep difficulties, with 9% reporting frequent occurrences (“often”, “most of the time”), which served as the strongest predictor of attrition. Further analysis observed that trainees with frequent sleep difficulties were 2.7 times more likely to be discharged (Taylor et al., 2020).

## 3 Role of chronic stress on maladaptation to military training: an allostatic load perspective

Given that a substantial relative incidence (∼60–65%) of overuse MSKIs, physical fitness decrements (∼30%), and worsened psychological wellbeing (∼10–25%) occur during military training, which may lead to consequences that threaten national security (Nindl et al., 2018; Good et al., 2020) and financial wellbeing of the US military healthcare system (Dijksma et al., 2020; Lovalekar et al., 2018; Pope et al., 1999), it is suggested that observed outcomes may be avoidable by the modification of risks. However, such risks must be delineated before prevention strategies can be implemented. Reductions in physical performance and the incidence of overuse MSKIs during training have been previously attributed to inadequate and excessive physical training stimuli, respectively (Burley et al., 2018), limited post-training recovery (Hansen et al., 2021), excessive mechanical loading on musculotendinous tissues (Feigel et al., 2023) and nutritional deficiencies (Alemany et al., 2008). Reduced psychological wellbeing, in turn, has been previously attributed to excessive or blunted stress responses (Tait et al., 2022; Taylor et al., 2017), introversion, or undesirable personalities (Saxon et al., 2020), childhood adversity (Ee et al., 2023), and a lack of previous training experience (Barrett et al., 2022). Though such factors have been revealed through the use of multi-factorial predictive models and their mitigation via individual interventions (Cooper et al., 2024), there is a lack of a unified physiological factor associated with such outcomes that may serve as the foundation bridging several psycho-physical outcomes and reduce the analytical burden in identifying risk factors.

Owing to an overall 12.5% rise in the recruitment rate of the US Armed Forces in the fiscal year of 2024 (US Department of Defense, 2025), structured military training programming functions to enable large masses of individuals to face similar external physical and psychological stress exposures, particularly under current gender-integrated physical training doctrine (Lovalekar et al., 2023). However, previous evidence reports a vast difference in the relative physiological response to stress, which may be detrimental to personnel experiencing greater stress exposures than their peers (Burley et al., 2018; Forse et al., 2024; O’Leary et al., 2018). Given research observing individualized maladaptive outcomes during training (Burley et al., 2018; Forse et al., 2024; Lovalekar et al., 2023), it is important to consider chronic activation of the physiological stress response perturbed by military training stressors, including energy restriction/deficits, sleep restriction/deprivation, physical overtraining, and cognitive distress, that may provoke allostatic load as an important contributor. The following section reviews the physiological response on neuroendocrine, immune, autonomic systems, its response to military training-related stressors and how these responses contribute to allostatic load.

### 3.1 The physiological stress response

*Stress* is defined as a constellation of events consisting of an external (i.e., environmental, psychosocial, mechanical) or internal (i.e., physical, biogenic) stimulus, whether actual or perceived, that precipitates a reaction in the brain followed by a physiological response nonspecific and specific to the stimulus to maintain homeostasis (Selye, 1950; Selye, 1976; Goldstein and Kopin, 2007; Nicolaides et al., 2015; Sher et al., 2020). In turn, *homeostasis* is characterized as the physiologic stability between interdependent biological systems (Billman, 2020). As such, stress encompasses an integrated definition to create a three-pronged construct: (i) a stressor (stimulus), (ii) a stress perception (detection and interpretation in the brain), and (iii) a stress response involving the activation of physiological fight-or-flight and neuroendocrine systems that serve as an adaptive mechanism to maintain homeostasis (Finnell et al., 2017; Gianaros et al., 2017; McEwen and Gianaros, 2011; Cohen et al., 2016; Schneiderman et al., 2005).

**FIGURE 3:**
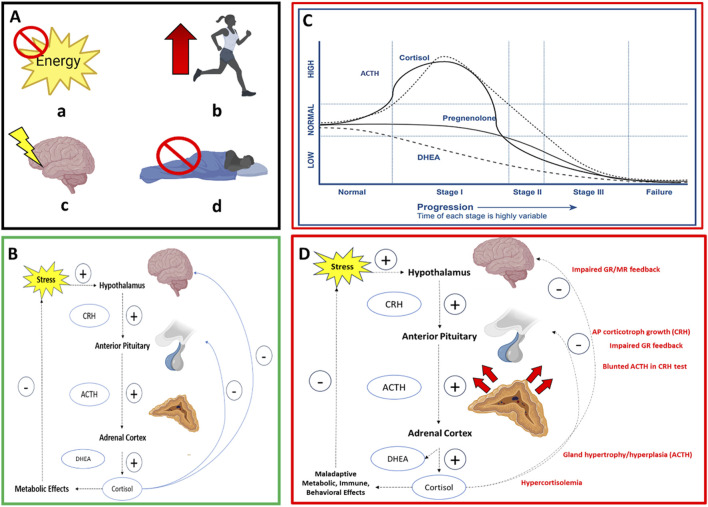
Box A illustrates four common military training-related stressors **(a)** energy restriction/deficiency **(b)** physical overexertion **(c)** cognitive stress **(d)** sleep restriction/deficiency, which are coined the Stressor Pool; Box B illustrates the HPA axis with negative feedback inhibiting CRH release from the hypothalamus and ACTH from the anterior pituitary gland. Box C illustrates the first model (Model 1) of HPA axis dysfunction and one mechanism of allostatic load wherein biomarker dynamics emulate Selye’s General Adaptation Syndrome model of overstimulated to an under-stimulated system (Stage I = Alarm Stage, Stage II = Resistance Stage, Stage III = Exhaustion Stage); CRH = corticotropic releasing hormone; ACTH = adrenocorticotropic hormone; DHEA = dehydroepiandrosterone. Box C illustrates the second model of HPA axis dysfunction and mechanism of allostatic load (Model 2) where initial hypercortisolism results in reduced glucocorticoid and mineralocorticoid receptor sensitivity on cells of the hypothalamus and anterior pituitary that impairs negative feedback and renders CRH and ACTH as growth factors. This results in larger functional masses capable of greater binding affinity to maintain the stress response over weeks.

The physiological stress response involves the integration of different brain regions and neuronal circuits responsible for the detection and interpretation of physical, psychological, or environmental stressors and the pro-survival and adaptive mechanisms that follow (McEwen and Gianaros, 2011; Cohen et al., 2016). Though diverse stressors engage distinct brain regions for processing and interpretation (Gianaros et al., 2017; McEwen and Gianaros, 2011), the initiation of the stress response involves the activation of the hypothalamic-pituitary-adrenal (HPA) and sympathetic-adrenal-medullary (SAM) axes for multi-system (i.e., immune, metabolic, cardiovascular) effects (Sher et al., 2020; Miller et al., 2007). Common military training stressors, including energy deficiency or restriction, physical overtraining, cognitive stress, and sleep deprivation/restriction (Figure 3A), have shown to activate the HPA axis and release its neuroendocrine factors for downstream influence on immune and autonomic activity (see Sections 3.2–3.5) by utilizing three primary structures (Figure 3B). The three structures that respond to such stressors include the paraventricular nucleus of the hypothalamus (PVN), the anterior pituitary gland, and the adrenal cortex. The PVN computes and integrates neuronal and humoral inputs to activate a specialized group of cells that control the level of activation of the HPA axis, including the regulation, synthesis, and secretion of corticotropin-releasing hormone (CRH) into the hypophyseal portal vasculature, which serves as a series of veins connecting two venous capillary beds for the transporting and exchanging of hormones between the hypothalamus and the anterior pituitary gland. The release of CRH and its subsequent binding to cognate receptors on corticotropes of the anterior pituitary can trigger the release of adrenocorticotropic hormone (ACTH) into the general circulation to bind to melanocortin-2 receptors on the surface of adrenal zona reticularis and the fasciculata cells of the adrenal gland. This binding triggers the release of glucocorticoids, including cortisol and dehydroepiandrosterone (DHEA), to the circulation (Figure 3B) (Nicolaides et al., 2015; Korte et al., 2005).

Cortisol, the primary glucocorticoid in humans (Oyola and Handa, 2017), binds to ubiquitous mineralocorticoid and glucocorticoid receptors on various body tissues to promote appropriate metabolic responses to environmental perturbations, including glycogenolysis and mobilization of free fatty acids for increased energy availability for expenditure and promote pro- and anti-inflammatory cytokine activation (Herman et al., 2012). DHEA, the glucocorticoid antagonist, serves to prevent excessive systemic inflammation and protects the neurologic machinery (i.e., glucocorticoid or mineralocorticoid receptors) from damaging effects of excess cortisol (Lennartsson et al., 2012; Lennartsson et al., 2022). Glucocorticoid receptor binding of hormones mediates an adaptive, negative feedback response inhibiting further stress hormone production at all levels of the HPA axis (McEwen and Gianaros, 2011; Herman et al., 2012) (Figure 3B).

In cohesion with the neuroendocrine response to stress, the immune system is triggered by the presence of inflammation reflected by circulating pro- and anti-inflammatory cytokine concentrations that mediate HPA axis activity via receptors in tissues associated with the axis (Kargl et al., 2024). Cytokines include mediators of the interleukin family (i.e., interleukin-6), tumor necrosis factor-alpha (TNF-α) (Turnbull and Rivier, 1995), and c-reactive protein (CRP). CRP, a systemic marker of inflammation, is closely linked to HPA activity, meaning that when CRP concentrations rise, the HPA axis also tends to activate, which can lead to increased cortisol and DHEA production (Sproston and Ashworth, 2018). When the immune system is triggered, pro-inflammatory cytokines are released, which can concomitantly stimulate the HPA axis to produce a bi-directional relationship with cortisol and DHEA. Anti-inflammatory cytokines also increase from this response as a negative feedback mechanism (i.e., interleukin-10) to counteract excessive pro-inflammatory effects (i.e., reactive oxygen species) (Kargl et al., 2024).

The physiological stress response also involves the activation of SAM axis owing to its neuronal synaptic circuitry relying on neurotransmitter communication between higher regions of the brain to peripheral receptors (Sapolsky et al., 2000). SAM activation involves the release of enzymes driven by sympathetic nervous system activity, such as salivary α-amylase (sAA), which is triggered by the release of norepinephrine and epinephrine and bind to β-adrenergic receptors in the salivary glands for its production. sAA is a reliable indicator of SAM activity (Nater and Rohleder, 2009), and has previously been used as a biomarker to assess the SAM response in ambient and military settings (Arhakis et al., 2013; Habersaat et al., 2018; Ishitobi et al., 2010; Klaus et al., 2019). The following sections aim to review empirical research of randomized controlled trial or observational cohort study designs that evaluated the influence of individual stressors commonly experienced during military training, including energy restriction or deficiency, physical overtraining or exertion, cognitive distress, and sleep deprivation or restriction, on the key drivers of allostatic load, including activity of the primary mediators of neuroendocrine, immune and autonomic systems (McEwen BS., 1998). Studies of healthy adults that assessed individual stressors and avoided confounding of additional stressors were included. Each summary included biomarkers commonly used in allostatic load research based on previous reviews (Juster et al., 2010; Carbone et al., 2022) to be consistent in the reporting of mediators across studies.

### 3.2 Role of energy deficit/restriction on the primary mediators of allostatic load

Energy restriction and/or deficits are commonly experienced during military training courses, with previous research reporting average deficits of 9.7 MJ/day over 8-days of military training (Alemany et al., 2008) and deficits ranging between 1000 and 4000 kcal d^-1^ over a 61-d US Army training course (Henning et al., 2014). Energy restriction or deficits during training have been attributed to restricted feeding times (Koltun et al., 2023), food choices, and periods of practiced energy restriction to simulate operations (Friedl et al., 2000). Koltun et al. observed that negative eating behaviors, such as energy restriction and deficits, are associated with worsened military health outcomes, including MSKI risk in both sexes (Koltun et al., 2023). A synopsis of the influence of energy deficit or restriction on activity of primary mediators of allostatic load in healthy, non-obese individuals can be shown in Table 1. From this synopsis, there are two main findings from the literature that may be drawn (Alemany et al., 2008; Henning et al., 2014; Ruffing et al., 2022; Degoutte et al., 2006; Huovinen et al., 2015; Buffenstein et al., 2000; Pasiakos et al., 2011) (Table 1).

**TABLE 1:** Empirical research of randomized controlled trial or observational cohort study designs that evaluated the influence of energy deficiency or restriction (independent variable) on primary mediators of allostatic load (dependent variable) in non-obese, healthy populations.

Author	Population	Methods	Outcome
Ruffing et al. (2022)	21 F Recreationally- Active	2 × 24 h blood sampling pre-post 3-mo ExRx + ER (−15%-60% EA, 5 ExRx∙wk^-1^ 70%–80% HR_max_)	**↔C**_**24hrAUC**_ **, ↔C**_**DaytimeAUC,**_ **↔C**_**mean**_ ,*↑C_**MorningAUC**_
Ackerman et al. (2013)	21 F AE vs.18 F EE vs. 20 F NE	Cross-sectional frequent sampling (2300–0700) to assess C (pulse amplitude, mass, half-life and AUC) dynamics	AE: *↑C_**SerumOvernightAUC**_ AE: *↑C_**SerumOvernightPulseAmp**_
Loucks et al. (1985)	9 F Balanced Diet vs. 9 F Restricted Diet vs. 8 F Control Sedentary	Frequent sampling (10-min intervals over 24 h) to assess C and ACTH dynamics following 10 kcal kg^-1^∙d^-1^ for 4-d	**Restricted Diet: ↔ACTH**_**PulseFreq;**_ **↔C**_PulseFrequency,_^*^ **↑C**_**24hUrine**_
Laughlin and Yen, (1996)	8 F, AA vs. 8 F, EA Trained Cyclists	Frequent sampling to assess 24-h dynamics of C	AA: *↑C_**Serum24hBasal**_
Alemany et al. (2008)	34 M US MOCs	9.7 MJ d^-1^ over 8-d	*↓DHEA_**SerumFreeBasalMean**_ **↔DHEA-S**_**SerumFreeBasalMean**_
Degoutte et al. (2006)	20 M Judoists	ER (-4 MJ d^-1^; n = 10) or No ER (n = 10) over 7-d	ER: *↑ACTH_**SerumBasalMean**_ , *↑C_**SerumBasalMean**_ ,*↓DHEA-S/C_**SerumBasalMean**_ , *↑DHEA-S_**SerumBasalMean**_
Henning et al. (2014)	23 M US Rangers	−1000–4000 kcal d^-1^ over 61-d	**↑C**_**SerumBasalMean**_ **, ↔DHEA**_**SerumBasalMean**_
Huovinen et al. (2015)	15 M Athletes	4-wk ER (HWR: −750 kcal d^-1^ w/≥2 g∙kg^-1^∙d^-1^ PRO (n = 8 M) or LWR: −300 kcal d^-1^ w/≥2 g∙kg^-1^∙d^-1^ PRO [n = 7 M])	**HWR: ↔C**_**SerumBasalMean**_ , *↑C_**HWRSerumBasalMean**_
Hooper et al. (2017)	9 M EHMC vs. 8 M NE	Cross-sectional sample of C dynamics between groups (EHMC: 27.2 ± 12.7 kcal d^-1^∙FFM^-1^; NE: 45.4 ± 18.2 kcal d^-1^∙FFM^-1)^	**EHMC: ↓C**_**SerumBasalMean**_
Pasiakos et al. (2011)	12 M, 1 F	48-h ER of <10% est. calorie requirements	*↑DHEA-S_**SerumBasalMean**_ , *↓C_**SerumBasalMean**_
Durguerian et al. (2018)	11 M Competitive Weightlifters	Diet Group (n = 6; −5% body mass over 6-d; 8.4 ± 3.6 MJ d^-1^) Control Group (n = 5; ±0% body mass over 6-d; 15.4 ± 5.0 MJ d^-1^)	Diet Group: *↑SAA_**SalivaBasalMean**_ **, ↔ C**_**SalivaBasalMean**_ **, ↔ DHEA**_**SalivaBasalMean**_
Waldman et al. (2020)	15 M Firefighters	4-wk ER (−25% of calories from baseline)	**↔C**_**SerumBasalMean**_ **, ↔CRP**_**SerumBasalMean**_ **, ↔SAA**_**SalivaBasalMean**_
Trevizol et al. (2019)	220 (153 F) Healthy Volunteers	2-y ER (−25% of calories from baseline; n = 145) or Control (n = 75) from CALERIE Trial	ER: *↓IL-6_**PlasmaBasalMean**_
Hennigar et al. (2021)	10 M Active-Duty Military Personnel	72-h SUSOPS w/Restricted Diet (−2047 kcal d ± 920 kcal d or −43% ± 9% energy deficit; n = 6) or 72-h SUSOPS w/Balanced Diet (+689 ± 852 kcal d or +18% ± 20% energy deficit)	Restricted Diet: *↑CRP_**SerumBasalMean**_ Both Groups: *↑IL-6_**PlasmaBasalMean;**_ *↑CRP_**SerumBasalMean**_

*Note*. Bolded values in Outcome column demonstrate a significant result (*p* < 0.05). C = cortisol; DHEA, dehydroepiandrosterone; DHEA-S, dehydroepiandrosterone-sulfate; ACTH, adrenocorticotrophic hormone; CRP = c-reactive protein; IL-6, interleukin-6; SAA, salivary α-amylase; F = female; M = male; MOCs, marine officer candidates; AE, amenorrheic exercisers; EE, eumenorrheic exercisers; NE, non-exercisers; ER, energy restriction; ED, energy deficit; EHMC, exercise-hypogonadal male condition; SUSOPS, sustained combat and training operations; HWR, high-weight-loss group; LWR, low-weight-loss group; PRO, protein; ExRx = exercise training program.

First, it is observed that neuroendocrine, autonomic, and immune biomarker concentrations depend on the relative severity (i.e., intensity) and duration of the energy restriction or deficit, such that the magnitude and length of the deficit propagates an inverted “U-shaped” curve where substantial restrictions or deficits in severity and/or prolonged duration promote acute increases in end-product concentrations followed by dampened responses with concentrations falling below baseline (Alemany et al., 2008; Henning et al., 2014; Ruffing et al., 2022; Degoutte et al., 2006; Buffenstein et al., 2000; Pasiakos et al., 2011). Energy deficits exemplified by active individuals consuming 4 MJ d^-1^ (<1000 kcal d^-1^) less than normal over 1 week (Degoutte et al., 2006) demonstrated significant increases in ACTH (+∼30%, *p* < 0.05), cortisol (+∼20%, *p* < 0.05) and DHEA (+5%, *p* < 0.05) and reductions in DHEA:Cortisol ratio (−20%, *p* < 0.001) from baseline (Degoutte et al., 2006). Over 2 days of near complete energy restriction where healthy individuals consumed less than 10% estimated calorie requirements, Pasiakos et al. (2011) (Pasiakos et al., 2011) observed a significant decrease in circulating cortisol (−70%, *p* < 0.001) and an upregulation of DHEA (+68%, *p* < 0.001) from baseline suggested to be driven by an inverse relationship with lower concentrations of leptin (Pasiakos et al., 2011). Likewise, Pritchard et al. (1999) (Pritchard et al., 1999) reported an 86% increase in dehydroepiandrosterone-sulfate (DHEA-S) following a 3-month energy deficit in healthy adult men (Pritchard et al., 1999). In contrast, however, Alemany et al. (2008) (Alemany et al., 2008) observed a significant reduction in DHEA concentrations following an 9.7 MJ d^-1^ energy deficit over an 8-day military training exercise (Alemany et al., 2008). However, as this training included additional stressors, this finding should be interpreted with caution.

Similar dose-response relationships are revealed concerning autonomic and immune biomarkers in response to energy restriction or deprivation (Durguerian et al., 2018; Hennigar et al., 2021). Among competitive weightlifters, Durgeurian et al. observed a significant increase in resting sAA (+364.60%, *p* < 0.05, Cohen’s *d* = 1.72) following a 6-day energy restricted diet of 8.4 ± 3.6 MJ d^-1^ when compared to a control group consuming 15.4 ± 5.0 MJ d^-1^ (Durguerian et al., 2018). However, after a 4-week energy restriction protocol with greater absolute severity (<25% of calories from baseline; 1650 ± 911 kcal d^-1^), Waldman et al. found no significant difference in resting sAA concentrations in firefighters (Waldman et al., 2020). Although Durgeurian et al. showed a conservative energy deficit (∼2007.65 kcal d^-1^) than Waldman et al. (∼1650 kcal d^-1^), the relative change in Durgeurian et al. (−1438 kcal d^-1^) was more severe than Waldman et al. (−400 kcal d^-1^), which may have contributed to the results (Table 1). Further, an acute 2-d energy restriction protocol has shown to evoke autonomic nervous system balance toward sympathetic dominance (Solianik and Sujeta, 2018), with more chronic energy deficits demonstrating a blunting in sympathetic activity measured by heart rate variability (Jenkins et al., 2022; Mazurak et al., 2011). During a 72-h sustained combat and training operations (SUSOPS) in active-duty personnel, Hennigar et al. observed a −43% energy deficit (−2047 kcal d^-1^) in a restricted diet group (2515 ± 171 kcal d^-1^) compared to an +18% energy deficit in a balanced diet group (5437 kcal d^-1^ ± 377 kcal d^-1^; *p* < 0.001). Further analysis^,^ observed significant increases following SUSOPS in CRP and IL-6 in both groups, with the restricted group showing a 59% greater increase in CRP than the balanced group (+2.6 ± 5.3 mg L^-1^, *p* < 0.001) (Hennigar et al., 2021). However, as the SUSOPS also included heavy physical training and sleep restriction (<4 h∙night^-1^), which may confound immune responses (Kargl et al., 2024), these results should be interpreted with caution. Over a 2-year energy restriction protocol, Trevizol et al. observed a significant reduction in IL-6 to indicate improvement in inflammation (Trevizol et al., 2019). Likewise, a 2020 meta-analysis demonstrated that energy-restricted diets reduce CRP concentrations compared to baseline through a considerable length of intervention (≥2 months) (Wang et al., 2020). In contrast, a 2022 systematic review observed an increase in circulating inflammatory cytokines in military personnel following field training exercises; however, this result may be confounded by additional stressors (E Silva et al., 2022).

Second, there is a general consensus that a reduction in body mass, as often observed following military training (Friedl et al., 2000; Nindl et al., 1997; Nindl et al., 2012), may serve as an indicator of increased basal HPA activity (Ruffing et al., 2022; Schorr and Miller, 2017; Villanueva et al., 1986). Ruffing et al. (2022) observed a significant correlation between reduced body mass and increased 24-h area-under-the-curve (AUC) cortisol concentrations (*r* = −0.473, *p* = 0.030), suggesting that cortisol could respond to reduced chronic energy stores (Ruffing et al., 2022). Additional studies observed increased basal cortisol concentrations among females with anorexia nervosa (Schorr and Miller, 2017; Misra and Klibanski, 2014) and in exercising females who were in a chronic energy deficit (Villanueva et al., 1986). Third, the hunger signal ghrelin may play a modulatory role in HPA axis activity during energy deficits (Misra et al., 2005). Previous studies observed a positive correlation between ghrelin and cortisol concentrations in females with anorexia nervosa (*r* = 0.480, *p* = 0.002) (Misra et al., 2005), with military-simulated energy restriction observing a negative association between satiety and cortisol (*r* = −0.550, *p* < 0.05) and DHEA-S concentrations (*r* = −0.620, *p* < 0.05) (Pasiakos et al., 2011). Hence, the augmented HPA activity during energy deficit or restriction appears to produce a dose-response relationship associated with deleterious changes in body composition and subjective satiety changes. Taken together, energy restriction or deprivation may serve as a potent driver of the primary mediators of allostatic load (Feigel et al., 2025a).

### 3.3 Role of physical overtraining on primary mediators of allostatic load

Physical overtraining–physical training conducted beyond one’s finite ability to recover adequately between training sessions and supported by reduced physical performance (Pope et al., 2018) - is commonly reported during military training (Nindl et al., 2017; Booth et al., 2006; O’Leary et al., 2018; Chicharro et al., 1998). Previous research reports that external training loads, characterized as the total work performed that contributes to the internal training load (Foster et al., 2017; Impellizzeri et al., 2019), which is defined as the relative physiologic indicator reflecting the psycho-physiological response to external loads (Foster et al., 2017; Impellizzeri et al., 2019), is often operationalized in military training by the total distance covered (O’Leary et al., 2018; Jurvelin et al., 2020; Whittle, 2022). Distances range, on average, between eight and twelve miles per day during initial training courses (Feigel et al., 2024a; Givens et al., 2023a; O’Leary et al., 2018) (Drain et al., 2015; Pihlainen et al., 2023). As a synopsis of the impact of physical overtraining, which may be experienced in military courses (Booth et al., 2006; O’Leary et al., 2018; Chicharro et al., 1998), on the primary mediators of allostatic load, four findings may be drawn (Henning et al., 2014; Roberts et al., 1993; Slivka et al., 2010; Fry et al., 1993; Volek et al., 2004; Cadegiani and Kater, 2017) (Table 2).

**TABLE 2:** Empirical research of randomized controlled trial or observational cohort study designs evaluating the influence of heavy physical training (independent variable) on primary mediators of allostatic load (dependent variable) in non-obese, healthy individuals.

Author	Population	Methods	Outcome
Roberts et al. (1993)	5 M Trained Runners	74-d of +100% training volume (mileage∙wk^-1^) from baseline with maintained self-reported intensity	*↑C_**SerumBasalMean**_
Slivka et al. (2010)	8 M Trained Cyclists	21-d of Cycling Tour (3,211 km) (169 km d^-1^ ± 4 km d^-1^) equivalent to +418% ± 142% training volume and +167.0 W ± 4.0 W (+47% ± 1%) of pre-training intensity	↔C_SalivaryBasalMean_, ↔T/C_SalivaryBasalMean_
Fry et al. (1993)	28 M Elite Weightlifters	1-wk of high-volume, full-body resistance training	*↓T/C_**SerumBasalMean,**_ *↓C_**SerumPostExRxMean**_
Volek et al. (2004)	17 M Resistance-Trained	4-week high volume, periodized full-body resistance training (week 1–2: high-volume, moderate intensity; week 3–4: moderate volume, high-intensity)	*↑C_**SerumBasalMean**_
Cadegiani and Kater (2017)	14 OTS Athletes, 25 Healthy Athletes, 12 Sedentary Controls	4-week high volume resistance training (5 days∙wk^-1^)	*↑C_**SerumBasalMean**_
Fernández-Garcia et al. (2002)	9 M Cyclists	3-wk “Vuelta a Espana” Cycling Tour	*↓C_**PlasmaPostBasalMean**_
O’Connor et al. (1989)	14 F Swimmers	4-wk of progressive high volume aerobic training (2,000 yd∙d^-1^–12,000 years d^-1^)	*↑C_**SalivaBasalMean**_
Bouget et al. (2006)	12 F Cyclists	4-d of +122% training volume and intensity	*↑C_**SalivaBasalMean**_ ; *↓DHEAS_**UrineBasalMean**_ **/C**_**SalivaBasalMean**_
Fry et al. (1994)	5 M Military Personnel of the Special Air Services Regiment of Australian Army	10-d twice-daily interval running sessions (morning: 15 × 1 min @18–21 km h^-1^ with 2 min rest between repetitions; afternoon: 10 × 1 min @18–21 km h^-1^ with 1 min rest between repetitions)	*↑IL-2_**SerumBasalMean**_
Tibana et al. (2016)	9 M CrossFit Trained	2-d of single ‘Workout of the Day’	*↑IL-6_**SerumBasalMean,**_ *↑IL-10_**SerumBasalMean,**_ *↓IL-10:IL-6_**SerumBasalMean**_
Tuan et al. (2008)	12 M Trained Runners	3-d of 30 min running exercise @85% VO2_max_	*↑TNF-α_**SerumBasalMean**_
Halson et al. (2003)	8 M Trained Cyclists	2-wk intensified cycling (14 ± 5 h∙wk^-1^ equal to +100% training volume of identical proportions of training intensity distribution from baseline)	↔TNF-α_PlasmaBasalMean_, ↔IL-6_PlasmaBasalMean_
Yasuda (2025)	21 F National-Level Athletes	10 months of volleyball training and competition (2–2.5 h d^-1^ for 6 days wk^-1^)	*↓SAA_**SalivaBasalMean**_
Chiodo et al. (2011)	16 (6 F) Taekwondo Athletes	1-d of Youth Taekwondo Competition	*↑C_**SalivaBasalMean**_ *↑SAA_**SalivaBasalMean**_
Collins et al. (2019)	21 M Team Sport Athletes	1 x Acute High-Intensity Functional Interval Training Session	*↑SAA_**SalivaBasalMean**_

*Note*. Bolded values in Outcome are significant (*p* < 0.05). C = cortisol; DHEA, dehydroepiandrosterone; DHEA-S, dehydroepiandrosterone-sulfate; T/C = testosterone to cortisol ratio; F = female; M = male; MOCs, marine officer candidates; AE, amenorrheic; EE, eumenorrheic; ER, energy restriction; ED, energy deficit; IL-2, interleukin-2; IL-10, interleukin-10; TNF-α, tumor-necrosis-factor-α; SAA, salivary α-amylase; W = watts; OTS, overtraining syndrome.

First, there is a general consensus that physical overtraining perturbs neuroendocrine activity such that it increases cortisol and DHEA concentrations as short as 1 week of training up to 74 days of a heavy physical training program (Roberts et al., 1993; Slivka et al., 2010; Volek et al., 2004; Cadegiani and Kater, 2017). However, this is not always observed in elite athlete populations owing to differences in physical fitness level and training experience (Slivka et al., 2010; Fernández-Garcia et al., 2002). Second, there are contradictions in results reported by individual variations in the stress response (Fry et al., 1993; Fernández-Garcia et al., 2002). For instance, the occurrence of hypocortisolism during heavy physical training (Fry et al., 1993; Fernández-Garcia et al., 2002) may be indicative of overreaching in some individuals wherein cortisol is blunted and positive adaptations cease (Armstrong et al., 2021). Third, the increase in HPA activity appears to depend on either heightened volume or intensity alone, but increased HPA activity may also be observed during high-volume, low-intensity training alone (Roberts et al., 1993; Hooper et al., 2017; O’Connor et al., 1989) or periodized high-volume, low-intensity training to low-volume, high-intensity training (Volek et al., 2004). Fourth, these observations appear independent of sex (Szivak et al., 2023a; Szivak et al., 2023b). Further, similar to the role of energy restriction (Table 1), heavy physical training demonstrates a dose-response relationship with increased relative severity or duration inducing a rise in neuroendocrine markers followed by a blunting effect (Fry et al., 1993; Fernández-Garcia et al., 2002; Bouget et al., 2006). However, as mentioned previously, differences in results may be attributed to physical fitness level (Slivka et al., 2010; Fernández-Garcia et al., 2002)

Fifth, physical overtraining has also been shown to alter inflammatory cytokine and autonomic biomarker concentrations at rest (Table 2). Fry et al. observed significant increases in markers of inflammation, including IL-2 (+183%, *p* < 0.001), following a 10-day, twice-daily, high-intensity interval running protocol in military personnel (Fry et al., 1993). Similarly, Tibana et al. showed significant increases in IL-6 (+99–197%), IL-10 (+14.4–21%) and a reduction in IL10:IL-6 ratio (−7.1%–8.9%) after a 2-day high-intensity functional interval training protocol (Tibana et al., 2018), and Tuan et al. observed significant increases in serum TNF-α (+∼100%, *p* < 0.05) following a 3-day intervention of 30-min running sessions at 85% VO2_max_ (Tuan et al., 2008). Previous research demonstrated that cytokines, including CRP, increases post-exercise (Tsao et al., 2009), with a peak around 24 h post-exercise (Reichel et al., 2020; Ispirlidis et al., 2008), but does not appear intensity-dependent (Tsao et al., 2009). Excessive resistance training can augment CRP concentrations in which may remain elevated up to 3 weeks (Fatouros et al., 2006). Concerning autonomic markers, Collins et al. (2019) (Collins et al., 2019) observed significant increases in sAA immediately after and 24-h post high-intensity exercise intervention in male athletes (Collins et al., 2019) and Chiodo et al. (2011) (Chiodo et al., 2011) observed significant increases following combat fighting competitions (Chiodo et al., 2011). Only one study to our knowledge investigated the chronic effects of a 10-month heavy physical training on sAA and observed a significant reduction in athletes indicating parasympathetic dominance and potential fatigue (−22%, p < 0.05) (Yasuda, 2025). Although sAA following chronic physical training remains largely uninvestigated, previous studies may support this finding by observing initial increases in sympathetic activity from heart rate variability (Plews et al., 2012) with a time-dependent reduction in sympathetic dominance toward parasympathetic dominance (Plews et al., 2012; Hynynen et al., 2006; Uusitalo et al., 1998; Flatt et al., 2017). Together, these data suggest physical overtraining may serve as a driver of the mediators of allostatic load.

### 3.4 Role of cognitive stress on primary mediators of allostatic load

Cognitive stress is the heightened perception of stress that can occur during military training (Conkright et al., 2022; Eddy et al., 2015) owing to physical training (Eddy et al., 2015), negative energy balance (Beckner et al., 2023), sleep deprivation (Passi et al., 2022), environmental conditions, and decision-making tasks (Conkright et al., 2022; Ben-Avraham et al., 2022). Newly recruited soldiers to mandatory military service face challenging psychological demands on a daily basis (Schei, 1994; Mažeikienė et al., 2021), including separation from family and friends, unpredictable and uncontrollable demands on their time, intense routines, and operating in a space laden with laws and hierarchies to contribute to cognitive stress (Boermans et al., 2013). Together, these factors can negatively affect the mental health of soldiers and increase the risk for attrition (Tait et al., 2022; Pope et al., 1999). Reduced cognitive performance may be reflected by cognitive fatigue (Eddy et al., 2015; Main et al., 2023), which can impact the ability to maintain an alert and attentive state and risk poor operational performance (Passi et al., 2022). Hockey (1997) (Hockey, 1997) observed that fatigue, in low controllability and high environmental demand situations, is related to HPA and SAM activation owing to direct engagement with an acute stressor (i.e., active problem-focused coping) (Hockey, 1997). Suarez and Perez found increased HPA activity in urban combat training when physical activity remained at a low level (Suárez and Pérez, 2013). Further analysis revealed cognitive stress caused by uncertainty regarding the location of threats, which led to fatigue and impaired post-combat information processing ability (Suárez and Pérez, 2013).

Table 3 summarizes a representative set of investigations (Henning et al., 2014; Lennartsson et al., 2012; Lennartsson et al., 2022; Budde et al., 2010; Schoofs and Wolf, 2011; Kudielka et al., 2004; Salvador et al., 2003) that assessed the influence of cognitive stress driven by the performance of cognitive tasks under time or duty constraints in occupational, classroom, or laboratory settings. Results of these studies observed significant increases (Lennartsson et al., 2012; Schoofs and Wolf, 2011; Kudielka et al., 2004; Salvador et al., 2003) or no change (Henning et al., 2014; Budde et al., 2010) in cortisol, DHEA, DHEA-S, or ACTH in response to a battery of cognitive assessments, including military training-related tasks (Conkright et al., 2022). Acute cognitive stress increases HPA activity among both sexes (Lennartsson et al., 2012; Schoofs and Wolf, 2011; Kudielka et al., 2004), whereas chronic cognitive stress can lead to blunted HPA activity (Miller et al., 2007). One meta-analysis observed a time-dependent HPA activity response with increased time since the onset leading to blunted morning and daily cortisol concentrations (Miller et al., 2007). However, it should be mentioned that Miller et al. observed that cognitive stress responses can be modulated by the nature (physical, social, traumatic), presence (morning, afternoon), emotional involvement (shame vs. loss), and controllability of the stressor (uncontrollable, controllable), which may affect neuroendocrine biomarker activity (Miller et al., 2007).

**TABLE 3:** Empirical research of randomized controlled trial or observational cohort study design evaluating the influence of cognitive stress (independent variable) on primary mediators of allostatic load (dependent variable) in non-obese, healthy individuals.

Author	Population	Methods	Outcome
Budde et al. (2010)	23 M, 17 F	Letter Digital Span and d2-test	↓C_SalivaryPostMean_
Schoofs and Wolf (2011)	39 M, 44 F	TSST or Placebo-TSST	M: *↑C_**SalivaryPostTSSTMean**_ ; F:*↑C_**SalivaryPostTSSTMean**_
Kudielka et al. (2004)	102 M and F	TSST	*↑ACTH_**PlasmaPostMean**_ ,*↑C_**SalivaryPostMean**_
Lennartsson et al. (2012)	20 M, 19 F	TSST	*↑ C_**SerumPostMean**_ ; *↑ ACTH_**PlasmaPostMean**_
Lennartsson et al. (2012)	20 M, 19 F	TSST	*↑ DHEA_**SerumPostMean**_ ; *↑ DHEA-S_**SerumPostMean**_
Salvador et al. (2003)	17 M Judoists	Pre-competition vs. practice salivary C dynamics	*↑ C_**SalivaryPreMean**_
Gouin et al. (2012)	55 Caregivers, 77 Controls	Cross-sectional blood sample	Caregivers: *↑ CRP_**SerumBasalMean,**_ *↑ IL-6_**SerumBasalMean**_
Giessing et al. (2020)	1 M Police Officer	3-week frequent saliva sampling (directly after waking, 30 min later, 6-h later, before bed) during work hours	↔SAA_SalivaBasalMean_
Wingenfeld et al. (2010)	215 (168 F) Nurses	13-h frequent sampling (07:00 h, 11:30 h, 17:30 h, 20:00 h) on a working day during an early shift	M: *↑SAA_**SalivaBasalMean,**_ F: *↑SAA_**SalivaBasalMean**_

*Note*. Bolded values in Outcome are significant (*p* < 0.05). C = cortisol; ACTH, adrenocorticotrophic hormone; DHEA, dehydroepiandrosterone; DHEA-S, dehydroepiandrosterone-sulfate; F = female; M = male; TSST, trier social stress test; SAA, salivary α-amylase; CRP = c-reactive protein; IL-6, interleukin-6.

Considering the influence of cognitive stress on immune and autonomic system function (Table 3), there is evidence of elevated circulating inflammatory markers owing to recurrent daily stressors (Gouin et al., 2012; Vineetha et al., 2014). Among a group of 53 chronic caregivers (56 months ±44 months) for dementia (≥5 h wk^-1^), Gouin et al. observed significantly higher concentrations of CRP and IL-6 compared to non-caregiving controls (Gouin et al., 2012). A systematic review on the influence of chronic occupational stress (i.e., employment, burnout and exhaustion, caregiver stress) identified elevated CRP concentrations than control groups (Johnson et al., 2013). However, previous research shows varied sAA responses to chronic stress (Habersaat et al., 2018; Giessing et al., 2020; Wingenfeld et al., 2010; Unno et al., 2013; Wood et al., 2021; Juster et al., 2011) despite robust increases during acute stress (Man et al., 2023; Knauft et al., 2021; Chacko et al., 2022; Teixeira et al., 2015). Individuals with chronic stress disorders demonstrate increases (Tanaka et al., 2012; Tanaka et al., 2013) or decreases in sAA (Teixeira et al., 2015; Wolf et al., 2008) during acute stressor tasks, but dampened basal sAA concentrations (Klaus et al., 2019; Wolf et al., 2008). Together, these data suggest cognitive stress may also perturb the mediators of allostatic load.

### 3.5 Role of sleep restriction or deprivation on primary mediators of allostatic load

Majority of military training studies observe that soldiers achieve less than the nightly recommended sleep duration of 7–8 h per night (Givens et al., 2023a; Kargl et al., 2024; Hansen et al., 2021), with most studies observing 4–6 h of sleep per night on average (Edgar et al., 2021; Givens et al., 2023a; Kargl et al., 2024; Taylor et al., 2020). Among a representative set of investigations assessing acute (1-2 nights) partial (4 h∙night^-1^) and total (0 h∙night^-1^) sleep deprivation on neuroendocrine function (Table 4), there is a general consensus of increased HPA activity across studies (Guyon et al., 2014; Balbo et al., 2010). Additional studies assessing the influence of semi-chronic (4 nights) or chronic (5+ nights) find blunted HPA activity owing to “psychological deactivation” or fatigue (Åkerstedt et al., 1982) and reduced HPA sensitivity (van Dalfsen and Markus, 2018). However, chronic short sleepers (<5 h∙night^-1^) have been shown to have elevated cortisol concentrations compared to normal sleepers, suggesting that the downregulation of the HPA axis may fail to occur in some individuals (Balbo et al., 2010).

**TABLE 4:** Empirical research selected owing to randomized controlled trial or observational cohort study design evaluating the influence of sleep deprivation (independent variable) on primary mediators of allostatic load (dependent variable) in non-obese, healthy individuals.

Author	Population	Methods	Outcome
Blumert et al. (2007)	9 M Weightlifter	1-d x TSD	↔C_SerumBasalMean_, ↔C_SerumPostExRxMean_, ↔T/C_SerumBasalMean_, ↔T/C_SerumPostExRxMean_
Guyon et al. (2014)	13 M Adults	2-d x PSD (4 h∙night^-1^)	*↑ACTH_**PlasmaBasalMean**_ ;* ↑C_**SerumBasalMean**_ **, ↔ACTH**_**PlasmaPulseFreqMean**_ **;↔C**_**SerumPulseFreqMean;**_ *↑ACTH_**PlasmaMorningMean**_ **;↔C**_**SerumMorningMean**_ *↑C_**SalivaryNightMean**_ ; *↑C_**SerumNightMean**_ **↔ACTH**_**PlasmaNightMean**_
Minkel et al. (2014)	14 M, 12 F Adults	1-d TSD (n = 12) vs. Normal (n = 14)	*↑C_**SerumBasalMean**_ ; *↑C_**SerumPostTSSTlMean**_
Akerstedt et al. (1980)	12 M Adults	2-d TSD	*↓C_**PlasmaBasalMean**_ ; *↓DHEA-S_**PlasmaBasalMean**_
Pajcin et al. (2017)	12 (5 F) Adults	2-d TSD	*↓SAA_**SalivaBasalMean**_

*Note*. Bolded values in Outcome are significant (p < 0.05). M = male; F = female; TSD, total sleep deprivation; PSD, partial sleep deprivation.

Investigations on the influence of sleep disturbance, defined as interruptions during periods of sleep, and deprivation has shown to alter immune and autonomic responses (Kargl et al., 2024; Irwin et al., 2016). A 2016 systematic review and meta-analysis (N = 72 studies) on the association between sleep disturbance, sleep duration, and inflammation in adults observed that sleep disturbance was associated with higher concentrations of CRP (ES 0.12; 95% CI = 0.05–0.19) and IL-6 (ES 0.20; 95% CI = 0.08–0.31), with shorter sleep duration, but not the extremity of short sleep, was associated with higher CRP (ES 0.09; 95% CI = 0.01–0.17) but not IL-6 (ES 0.03; 95% CI: −0.09–0.14). However, neither sleep disturbances nor sleep duration was associated with TNF-α (Irwin et al., 2016). Among salivary markers, recent literature suggests that sAA may be a cross-species marker of sleep deprivation in tactical (i.e., first responder) and military populations (Lindsey et al., 2025) and individuals with sleep disorders (Thieux et al., 2024). Pajcin et al. observed the influence of 2-d TSD (50-h) on sAA and observed a significant diurnal profile wherein concentrations increased throughout the morning and afternoon (*p* < 0.001) and steadily declined in the evening and early-morning (*p* < 0.001). These results suggested that sAA may be sensitive to the diurnal rhythm for arousal and tracking the diurnal drive for alertness (Pajcin et al., 2017) (Table 4). However, there does not appear to be a consensus as to the direction of sAA with subjective feelings of sleep disturbance and repercussions of sleep debt (i.e., sleepiness and cognitive performance) (Thieux et al., 2024).

### 3.6 Mechanism of allostatic load from military training-related stressors

Although military training-related stressors do not occur in isolation during training (Nindl et al., 2018; Friedl et al., 2000), and exhibit bi-directional effects, such as sleep deprivation affecting subjective feelings of psychological stress (Schwarz et al., 2018), the above findings may reveal a pattern of the influence of stressors on primary mediators of allostatic load by demonstrating acute increases and chronic reductions in biomarker concentrations during stress (Tables 1–4). Chronic stress can lead to one or more forms of HPA axis dysfunction (Figures 3C,D) and alter immune and autonomic system function, which may serve as a mechanism of allostatic load (Selye, 1950; van Dalfsen and Markus, 2018; Karin et al., 2020) as biomarker concentrations may fall within ‘at-risk’ bounds to support the computation of the ALI (Juster et al., 2010).

*HPA axis dysfunction* is characterized as a dynamic compensatory response to chronic stress that begins with initial hypercortisolism followed by hypocortisolism (Fries et al., 2005) or diurnal dysrhythmia (McEwen, 2007). The mechanism of HPA dysregulation derives from an evoked HPA axis that engenders an over-responsive system (e.g., hypercortisolism) toward an under-responsive or non-responsive system (e.g., hypocortisolism) (Selye, 1950). Progression from an over to under-responsive system is reflected by one or more forms of HPA axis dysregulation leading to allostatic load (McEwen, 2007; Juster et al., 2011) (Figures 3C,D). Model 1 is one form that illustrates the dynamics of neuroendocrine biomarker responses broken down into three stages when under the influence of chronic stress. These three stages illustrate the trajectory of biomarker concentrations and emulate Han’s Selye General Adaptation Syndrome (GAS) theory (Selye, 1950) (Figure 3C). The first stage of biomarker activity is the “Alarm Stage”, described as an acute, adaptive response to a stressor. This can also be illustrated by the allostasis model (Figure 1), where a response appropriately meets a demand observed by increased cortisol, DHEA, and ACTH concentrations (Korte et al., 2005). If the stress continues, however, the ‘Resistance Stage’ occurs, which is characterized by chronic activation of the physiological stress response (“chronic allostasis”) as observed by heightened biomarker concentrations. Notably, this stage risks degradation of protective negative feedback mechanisms (i.e., deterioration of glucocorticoid receptor sensitivity) or *primary outcomes,* as shown in the allostatic load model (Figure 2A). Finally, if the stress continues, the “Exhaustion Stage” occurs, which is characterized by a reduction in circulating biomarker concentrations that can signal allostatic load (Sher et al., 2020). Taken together, Model 1 reports that under chronic stress, ACTH will drive cortisol production and deplete DHEA production that leads to reduced cortisol production toward hypocortisolism in the Exhaustion Stage (Stephens and Wand, 2012). Hence, this model benefits the prediction of neuroendocrine biomarker trajectories and downstream immune and autonomic biomarker trajectories to complement the aforementioned results of empirical research on common military training-related stressors (Tables 1–4). Hence, Model 1 may serve as one mechanism leading to allostatic load (McEwen BS., 1998; Sher et al., 2020).

A second mechanism of allostatic load is shown in Model 2 (Figure 3D). Model 2 purports that HPA dysfunction and the downstream effects of immune and autonomic function arise from changes in the total functional masses of the HPA hormone-secreting glands including the adrenal cortex and anterior pituitary (Karin et al., 2020). Karin et al. reported that this mechanism occurs from an initial hypercortisolism followed by reduced DHEA production and glucocorticoid receptor and mineralocorticoid receptor sensitivity of the cells in the hypothalamus and anterior pituitary that impair the negative feedback loop of the HPA axis (Karin et al., 2020). Reduced cell receptor sensitivity with stress hormone production may render the hormones of the HPA as growth factors for the glands in the axis where excessive secretion of CRH can drive pituitary corticotroph cell growth and ACTH can drive adrenal gland hypertrophy/hyperplasia to result in larger functional masses capable of greater binding affinity to maintain the stress response over weeks. Consequently, this form of HPA axis dysfunction can lead to a similar over-to-under responsive neuroendocrine, immune, and autonomic nervous system activity (Henning et al., 2014; Kargl et al., 2024; Karin et al., 2020) as observed in response to military training-related stressors (Tables 1–4). Hence, Model 2 may serve as a second mechanism of allostatic load. Owing to the role of military training-related stressors on allostatic load, the next section summarizes the impact of allostatic load quantified by ALI on physical performance and psychological and musculoskeletal health, in non-obese, healthy adults and, where available, military personnel.

## 4 Impact of allostatic load on physical performance, psychological, and musculoskeletal health

### 4.1 Physical performance

Previous evidence observes that ALI is negatively associated with physical performance outcomes assessed by a battery of maximal strength and balance assessments (Germano et al., 2023; Hansen et al., 2016). Among 1101 healthy volunteers (65–74 years), Germano et al. observed ALI was inversely associated with score on the Short Physical Performance Battery (SPPB) score (β = −0.234, *p* < 0.001), with indirect effects evidenced between age and socioeconomic status (Germano et al., 2023). Similarly, among 5467 healthy volunteers (48–62 years), Hansen et al. found that the ALI mediated the association between education and physical performance (chair rise ability, postural balance, sagittal flexibility) and muscle strength (jump height, trunk extension, and flexion, handgrip strength) and accounted for 2%–30% of the total effect among women (Hansen et al., 2016). As a secondary analysis of data from the MacArthur Studies of Successful Aging study, Seeman et al. observed step-wise reductions in similar physical performance outcomes with every 1-unit increase in ALI (Seeman et al., 1997) and during a follow-up of 7 years (Seeman et al., 2001). Seeman et al. also found the ALI outperformed predicting physical dysfunction to a greater degree than its individual sub-components, suggesting its benefit of determining risk of physical performance decline (Seeman et al., 2001). However, these investigations were conducted among older (mid to late-life) adult populations (Germano et al., 2023; Hansen et al., 2016). Nevertheless, recent research has linked high ALI with worsened physical performance in younger (<40 years) populations and in military personnel (Feigel et al., 2025a; Hastings et al., 2022). Feigel et al. observed a significant negative association between change (Δ) in ALI from baseline and change in physical performance in men from elements of the USMC Physical Fitness Test (PFT) (ΔPullups: β = −0.88, R^2^ = 0.60, 95% CI: −1.55, −0.21; ΔPush-Pull PFT Score: β = −2.87, R^2^ = 0.60, 95% CI: 4.99, −0.75; Δ Total PFT Score: β = −3.48, R^2^ = 0.58, 95% CI: −5.76, −1.19) to suggest a potential role of chronic stress on military physical performance (Feigel et al., 2025a). Data from the National Survey of Midlife Development in the United States (N = 2055, 26–86 years) demonstrate a negative association with ALI and physical function (grip strength: β = −0.11, 95% CI: −0.15, −0.07; gait speed: β = −0.20, 95% CI: −0.24, −0.16) (Hastings et al., 2022). Although military training studies lack use of the ALI, previous research demonstrates an association between altered neuroendocrine and autonomic hormone profiles following training and worsened physical fitness characteristics, such as muscular power and strength (Szivak et al., 2018), which are important attributes for occupational task performance (Feigel et al., 2024b). Further evidence suggests that increased inflammatory cytokine concentrations can hinder muscle protein synthesis of lean muscle mass (Miller et al., 2022) and negatively influence upper and lower-body muscular strength (Sharma Ghimire et al., 2023). Hence, further research of ALI on physical performance in in-training personnel is warranted.

### 4.2 Psychological wellbeing

Previous epidemiological studies demonstrate a positive association between ALI score and symptoms of reduced psychological wellbeing in healthy individuals (Guidi et al., 2020), including anxiety (Gou et al., 2025; D’Alessio et al., 2020), depression (Gou et al., 2025; D’Alessio et al., 2020), and perceived stress (Guidi et al., 2020; Beckie et al., 2016; Juster et al., 2010; Geronimus et al., 2006) and a negative association with resilience (Felix et al., 2023), all of which are reported during military training (Forse et al., 2024; Bulmer et al., 2022b; Taylor et al., 2009; Guo et al., 2021). However, cognitive reappraisal was indirectly associated with lower ALI, whereas the tendency to use emotion suppression was indirectly associated with greater ALI (Ellis et al., 2019). Sleep health has been tied to allostatic load from a systematic review demonstrating a positive relationship between chronic sleep difficulty level and ALI (Christensen et al., 2022). Guidi et al. observed poorer objective and subjective sleep quality was associated with ALI in four separate investigations (Guidi et al., 2020). A 2022 systematic review and meta-analysis by Christensen et al. observed a significant negative association between sleep health, characterized by sleep duration and sleep quality (i.e., greater time in restorative sleep stages as compensation) on ALI among epidemiological studies. However, it was also reported that sleep may be bi-directional where poorer sleep quality may contribute to ALI if sleep health is not improved with intervention (Christensen et al., 2022). Interestingly, recent work from our group observed a significant negative association between Δ in sleeping difficulty level and Δ ALI by the end of a 10-week military training course in both sexes (ΔSD: β = −1.25 to −0.56, R^2^ = 0.35 to 0.82, p < 0.001–0.046) (Feigel et al., 2025a). Further research into the influence of ALI on psychological wellbeing outcomes in military training populations is also warranted.

### 4.3 Musculoskeletal health

Gallagher and Barbe (2022) examined the role of allostatic load on musculoskeletal disorders, characterized as injury, dysfunction, or impairment to the muscle, ligament, tendon, or bone, during occupational settings (Gallagher and Barbe, 2022). Their findings suggested that musculoskeletal disorders, including overuse MSKIs, may result from impaired tissue repair mechanisms driven by mechanical stress. These impairments, in turn, are influenced by underlying inflammatory, autonomic, and neuroendocrine dysfunctions associated with allostatic load, which is precipitated by chronic psychological and physical stress. Overuse MSKIs are comprised of inflammatory and degenerative conditions in musculoskeletal tissues involving muscles, tendons, ligaments, and peripheral nerves (Edwards, 2018). The authors found that physical work risk factors for overuse MSKIs included high force demands, repetitive work, adoption of non-neutral postures, and repeated heavy lifting, with a combination of high psychosocial work demands during work settings (da Costa and Vieira, 2010), which are common attributes experienced during military training courses (Vaara et al., 2022). Additional psychological risk factors included perceived stress at work, psychological job demands, and low job control (Bongers et al., 1993; Bongers et al., 1993; Deeney and O’Sullivan, 2009). Together, the authors purported that the presence of psychological and physical stress may lead to allostatic load and overuse MSKI owing to a slower-than-normal healing response in the tissue. This may result in faster damage development in musculoskeletal tissues and higher overuse MSKI risk (Gallagher and Barbe, 2022).

Epidemiological studies demonstrate relationships between ALI and musculoskeletal disorders (Guidi et al., 2020; Mori et al., 2014). Among 703 healthy men and women (median age: 56), mixed-effects linear regression controlling for clustering within families and adjusted for age, gender, race/ethnicity, body mass index, menopausal transition stage, childhood socioeconomic status, adult finances, education level, and study center, each standard deviation increment in ALI was associated with between 0.10 and 0.11 standard deviation decrements in lumbar spine bone mineral density (all *p* < 0.05) (Mori et al., 2014). Symptom frequency and intensity were associated with higher ALI among chronic fatigue syndrome patients compared to controls (Maloney et al., 2006; Goertzel et al., 2006). Further research on the influence of ALI on musculoskeletal health on training personnel is warranted (Feigel et al., 2024a).

## 5 Future of allostatic load assessment in military training research: consideration of commercial wearable devices for monitoring verified digital phenotypes of allostatic load

Owing to the influence of military training-related stress on the primary mediators of allostatic load (Table 1–4), and empirical research of allostatic load on physical performance and psychological and musculoskeletal health, there is a growing support for the role of allostatic load on military training-related maladaptive outcomes (Feigel et al., 2024a; Feigel et al., 2025a). However, further empirical research is warranted in this area to support these findings. Therefore, the future of allostatic load monitoring in military training research may direct toward two options for its measurement: the ALI method (Feigel et al., 2025a) or commercial wearable-based methods (Feigel et al., 2024a).

Although the ALI offers the advantages of understanding the biological process underpinning cognitive and physical dysfunction in response to chronic stress when studied under longitudinal study designs, such as using sample-specific biomarker cut-off values for increased specificity (Juster et al., 2011), and selecting biomarkers relevant to the target population and stress exposure (Karlamangla et al., 2002; Mauss and Jarczok, 2021), the ALI has limitations (McLoughlin et al., 2020; Carbone et al., 2022; Magtibay and Umapathy, 2023). First, longitudinal tracking of ALI requires more than one blood draw or salivary sample (Figure 2B), which increases participant burden, risk of missing data, and analytical complexity, especially when up to 20 biomarkers are included (Juster et al., 2010). Second, there have been inconsistent ALIs used in the literature comprising different biomarkers (Juster et al., 2010) and algorithms for its computation to limit replication (Carbone et al., 2022). Hence, for occupational populations where conducting repeated ALI assessments presents the logistical challenges of obtaining more than one blood draw or salivary sample, such as military personnel, Magtibay and Umapathy proposed that ubiquitous, commercial wearables (Figure 2C) may overcome challenges (Magtibay and Umapathy, 2023).

**FIGURE 4:**
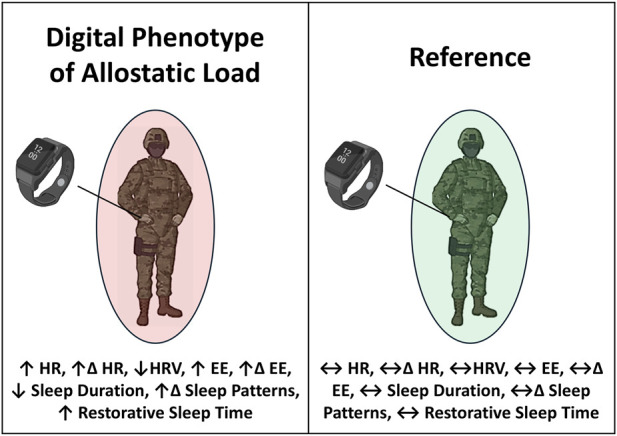
The digital phenotype of allostatic load may be characterized by one or more digital signatures, including chronically elevated and variable cardiometabolic activity, blunted heart rate variability, and altered neurobehavioral (i.e., sleep health) patterns in response to chronic occupational stress. Digital signatures of the phenotype can be detected by continuously worn, wrist-worn commercial wearable devices when compared to a reference not exposed to chronic occupational stress. HR = heart rate, EE = energy expenditure, HRV = heart rate variability. Δ = Day-to-Day Change; ↑ = increase from normal; ↔ = no change from normal; ↓ = decrease from normal.

Through continuous monitoring, commercial wearable devices use signal features, such as photoplethysmography (PPG) and accelerometry, to capture downstream cardiometabolic and neurobehavioral responses (i.e., sleep architecture or behavior) perturbed by chronic stress (Feigel et al., 2024a; Friedl, 2018). This approach has been suggested to complement the ALI where combinations of wearable-derived signals may define a wearable-derived “digital phenotype” of allostatic load (Feigel et al., 2024a; Corrigan et al., 2021). This phenotype may be characterized by one or more digital signatures, including chronically elevated and variable cardiometabolic activity, reduced heart rate variability, and/or altered sleep architecture—such as increased time spent in restorative sleep stages—in response to chronic occupational stress (Magtibay and Umapathy, 2023) (Figure 4). Our group provided empirical support for this phenotype identified using continuous monitoring of commercial wearable devices in military personnel who experienced tertiary outcomes of allostatic load, including overuse MSKI (Feigel et al., 2024a). Together, these findings, as well as the technological advances in sensors, may support commercial wearable devices as a promising approach (Figure 2C) to detect downstream cardiometabolic and neurobehavioral effects (secondary outcomes; Figure 2A) and assess allostatic load in-the-field (Magtibay and Umapathy, 2023).

Use of physiological time-series data from continuously worn commercial (i.e., wrist-worn, durable, low-burden) wearable technology for allostatic load assessment remains understudied (Feigel et al., 2024a; Corrigan et al., 2021). Using allostasis and allostatic load models as tools for measuring stress responses in occupational settings, Magtibay and Umapathy reported that low-burden, wrist-worn wearable devices within a robust human-machine learning framework could be useful to measure *digital biomarkers* of allostasis, characterized as cardiovascular, metabolic, and behavioral responses to everyday life, and use those signals to detect physiological characteristics of allostatic load*,* which may take the form of a digital phenotype (Magtibay and Umapathy, 2023) (Figure 4). The authors also reported signals from wearables, such as PPG, triaxial accelerometry, and thermometry, could indicate stress-induced autonomic responses activated by HPA and SAM axes (Magtibay and Umapathy, 2023). Additionally, data features captured by most commercial wrist-worn wearables could be used to determine its relationship with ALI as an alternative proxy of allostatic load assessment in occupational settings (Magtibay and Umapathy, 2023). Software algorithms could reveal physiologic responses and behavioral tendencies owing to allostatic load, such as altered sleep patterns (Christensen et al., 2022). Previous research suggests that wrist-worn devices may improve participant compliance than waist-worn devices (Kim et al., 2019; Wolpern et al., 2019) and promote continuous monitoring without obstruction or interference during military training (Feigel et al., 2024a; Friedl, 2018; Hinde et al., 2021). As the use of the ALI increases in military training research (Feigel et al., 2025a), the employment of commercial wrist-worn wearable devices (Hinde et al., 2021; Feigel et al., 2025b; Friedl and Looney, 2023) may provide the opportunity to determine verifiable, wearable-derived signals of personnel experiencing allostatic load.

However, an important limitation found in the literature when using wearables to detect allostatic load (Corrigan et al., 2021; Corrigan et al., 2023) is the lack of aligning the signals with the formal definition of allostatic load as proposed by McEwen and Stellar (McEwen, 1993). Based on the definition, the threshold by which allostatic load occurs is not immediately informative with only independent variables (i.e., physiological signals of stress). However, the distinction when allostatic load is experienced may be revealed through the presence of (i) whether maladaptive psycho-physiological outcomes (secondary or tertiary outcomes; dependent variables) are associated with the signals (McEwen, 1993; Magtibay and Umapathy, 2023) or (ii) signals are associated with ALI (Magtibay and Umapathy, 2023). Allostatic load is a biological state where physiological stability fails owing to multi-system dysregulation and occurs concomitantly with secondary or tertiary outcomes (McEwen, 1993). The ALI has been validated through the observation of its association with secondary and tertiary outcomes (Seeman et al., 1997; Seeman et al., 2001), and associated with high ALI scores (i.e., ALI >3 or 4) (Juster et al., 2010; McLoughlin et al., 2020). Previous studies reveal the utility of commercial wearables in the detection of acute (Chen et al., 2017; Cho et al., 2019; de Vries et al., 2022; Erickson et al., 2022), and chronic occupational stress (O’Leary et al., 2018; Hinde et al., 2021; Erickson et al., 2022; Wyss et al., 2014). However, there have been very few studies that adopted this theory-based ruling to use commercial wearables and determine whether allostatic load is experienced (Feigel et al., 2024a). There remains a lack of research assessing the relation between digital signals and high allostatic load, and with secondary or tertiary outcomes (Feigel et al., 2024a). Such findings may advance the use of commercial, wrist-worn wearables in-the-field for high allostatic load risk detection in military personnel. The next section summarizes the empirical research that used wearable-based physiological signals for evaluating allostatic load detection in the literature thus far (commercial and non-commercial).

### 5.1 Physiological characteristics of allostatic load

#### 5.1.1 Chronically elevated and variable heart rate

Among the first studies employing wearable devices to evaluate stress system perturbations using allostatic load as a framework, Milosevic et al. developed a research methodology to examine the real-time multi-modal responses to work stress of fifteen nursing volunteers conducting a representative protocol during a workday (Milosevic et al., 2013). They monitored physiological parameters of beat-to-beat blood pressure, heart rate, heart rate variability, respiratory rate, and galvanic skin resistance during a simulated, high-fidelity patient simulator (30-min) intervention (tracheostomy protocol with and without respiratory distress) through the use of garment-worn and electrode-based sensors attached on the chest. Repeated-measures analysis of variance evaluated the physiological changes before, during, and after the protocol, and revealed a significant strain placed on volunteers during the test, owing to a significant rise in blood pressure, heart rate, respiratory rate, and reduction in heart rate variability, with some participants returning to baseline after the test. The authors suggested that this response may be characteristic of allostatic load (Milosevic et al., 2013). However, the ALI, secondary, or tertiary outcomes were not assessed, thus hindering the verification of chronically elevated heart rate and lower heart rate variability as a digital signatures of allostatic load. Recent research from our group, however, observed that these digital signatures measured by valid and reliable commercial wrist-worn devices, were associated with a tertiary outcome of allostatic load including overuse MSKI occurrence (Feigel et al., 2024a). Although the ALI was not assessed from our group in the aforementioned study (Feigel et al., 2024a), this finding may support these signals of the digital phenotype (Feigel et al., 2024a) (Figure 4). Nevertheless, further research on the relationship between these signals and ALI is warranted.

#### 5.1.2 Chronically elevated and variable energy expenditure

Allostasis and allostatic load cost metabolic energy for responding and attempting to adapt to environmental and physiological perturbations to maintain homeostasis (Bobba-Alves et al., 2022). Bobba-Alves et al. reported that the transition from allostasis to allostatic load can be defined by an energetic tradeoff wherein allostasis and stress-related energy costs compete with growth, maintenance, and repair mechanisms (Bobba-Alves et al., 2022). However, empirical research using wearable-based energy expenditure as a digital signature to determine whether allostatic load is experienced in personnel remains limited. Givens et al. employed a wrist-worn device for continuous physiological monitoring over a 10-week USMC training course and observed elevated total daily energy expenditures, on average, of 3000 kcal∙day^-1^ (Givens et al., 2023a). Using the allostatic load framework, Feigel et al. observed that male and female personnel who sustained an overuse MSKI had chronically elevated and variable wrist-worn commercial wearable-derived energy expenditure compared to uninjured counterparts, which may help verify this digital signature of allostatic load (Feigel et al., 2024a). To our knowledge, this was the only investigation that used the allostatic load model to evaluate whether wearable-derived energy expenditure was associated with a tertiary outcome. Although these results may suggest that chronically elevated and variable energy expenditures may be digital signatures of the phenotype (Figure 4), further research on the relationship between wearable-derived energy expenditure and ALI is warranted.

#### 5.1.3 Altered sleep behavior

Altered sleep behavior is reported as a consequence of allostatic load owing to elevated neuroendocrine, inflammatory, and autonomic nervous system activity experienced from daytime stressors (McEwen, 2006; Christensen et al., 2022; Balbo et al., 2010). Magtibay and Umapathy report that wearables may capture sleep health for allostatic load monitoring (Magtibay and Umapathy, 2023). Fortunately, previous research supports this claim. Using a 23-biomarker ALI, Bei et al. assessed the association of ALI and wearable-derived sleep health via actigraphy, including bed time, rise time, sleep efficiency [ratio of total sleep time to time in bed, multiplied by 100 to yield a percentage], total sleep time, sleep onset latency, wake-after-sleep-onset, and observed that later average bed time (β = 0.15, SE = 0.02, 95% CI: 0.03, 0.26) and shorter average total sleep time (β = −0.13, SE = 0.02, 95% CI: −0.24, −0.02) were associated with a higher ALI score, and more variable sleep-onset-latency (β = 0.14, SE = 0.02, 95% CI: 0.02, 0.26) and wake-after-sleep-onset (β = 0.13, SE = 0.04, 95% CI: 0.01, 0.26) were associated with ALI (Bei et al., 2017). Martucci et al. observed no significant difference in an 11-biomarker ALI score between patients with insomnia confirmed via actigraphy and normal sleepers (insomnia: 2.5 ± 1 vs. normal: 2.0 ± 1, *p* = 0.200) (Martucci et al., 2020). However, Feigel et al. found that wrist-worn device-derived absolute and relative time spent in restorative sleep stages, including deep and light stage sleep, was associated with overuse MSKI status during military training (Feigel et al., 2024a). Together, these results may suggest that shorter sleep time, inconsistent sleep patterns, and greater time spent in restorative sleep may serve as signatures of the phenotype (Figure 4).

#### 5.1.4 Blunted heart rate variability

Corrigan et al. (2021) conducted a systematic review on the influence of military training or tactical operator stress on heart rate variability as a method to assess allostatic load (Corrigan et al., 2021). However, whether the signals were associated with their formal established measurement, the ALI, or secondary or tertiary outcomes were not reported to support the use of heart rate variability as a measure of allostatic load. Nevertheless, the authors revealed an overall reduction in heart rate variability indices in response to acute physical and cognitive stressors, with slower rates of recovery after the completion of acute occupational stressors that was dependent on the magnitude of the stress, as well as chronic stressors observed during nightly heart rate variability assessments (Corrigan et al., 2021). Only one study assessed daily resting heart rate variability in soldiers with markers of stress to provide context to the signal responses (Huovinen et al., 2009). However, without linking the signals with its formal measure or secondary or tertiary outcomes, the definition of allostatic load may be neglected its full use in explaining psycho-physiological maladaptation. Further investigation linking heart rate variability, ALI, and psycho-physical outcomes is warranted. Hence, further research on whether blunted heart rate variability may serve as a digital signature of the phenotype is warranted (Figure 4). A summary of the wearable-derived signals used for allostatic load assessment in the literature, their definitions, and signals often observed in consumer, wrist-worn commercial wearable devices based on Peake et al. (2018), which may be useful for allostatic load measurement in military training environments, is found in Table 5.

**TABLE 5:** Description of existing wearable-based measures of allostatic load used in the literature and measures that are programmed in modern wrist-worn, commercial wearables from Peake et al. (2018).

Measure	Description	Available in most wrist-worn, commercial wearables
Heart rate (bpm) (Milosevic et al., 2013; Bulmer et al., 2022a)	Rate of cardiac contractility per minute; Rate reflects dominance of autonomic nervous system activity with lower rates indicative of parasympathetic branch activation and higher rates indicative of sympathetic branch activity; Sensitive to HPA and SAM activity from stress. Available in most commercial wearables	Yes
Heart rate variability (Milosevic et al., 2013; Corrigan et al., 2023)	Period (time) between beats (i.e., inter-beat interval duration) measured by frequency or time-domain methods. Nonlinear time series analysis methods (i.e., detrended fluctuation analysis) demonstrate utility in reflecting frequency domain methods to stress. Higher variability has been associated with greater autonomic nervous system balance between branches, with lower variability associated with singular branch dominance that may require heart rate for context. Not available in most commercial wearables	Yes (if No, calculate from time-series data)
Total daily energy expenditure (kcal, rate) (Magtibay and Umapathy, 2023)	Magnitude or rate of total daily calories burned that is estimated using a combination of heart rate, anthropometric, demographic, and activity data captured by wearable devices. Available in most commercial wearables	Yes
Physical activity energy expenditure (kcal, rate) (Magtibay and Umapathy, 2023)	Magnitude or rate of total calories burned during physical activity estimated by wearable devices	Yes
Systolic blood pressure (mmHg) (Milosevic et al., 2013)	Pressure in the arteries when the heart beats and pumps blood throughout the body; Measured by a noninvasive 24-h ambulatory blood pressure monitor worn on the peripheral upper limb to provide a real-time picture of blood pressure fluctuations during the day and evening	No
Diastolic blood pressure (mmHg) (Milosevic et al., 2013)	Pressure in the arteries when the heart relaxes between beats; Measured by a noninvasive 24-h ambulatory blood pressure monitor worn on the peripheral upper limb to provide a real-time picture of blood pressure fluctuations during the day and evening	No
Electrodermal activity (Milosevic et al., 2013)	Changes in the skin’s electrical conductance due to sweat gland activity, which is a non-invasive method to assess emotional and physiological arousal to stress; Sensitive to metabolic and neuroendocrine systems; Measured by applying a small current between two electrodes placed on the skin and measuring the skin’s resistance or conductance to that current (microsimens). Increased activity is associated with elevated sympathetic nervous system activity	No
Sleep Duration (hh:mm:ss) (Bulmer et al., 2022a; Christensen et al., 2022)	Time period at which one is asleep, with lesser time often associated with indices of allostatic load from biomarker-based measurements	Yes
Sleep Architecture (hh:mm:ss, %) (Christensen et al., 2022)	Time or proportion spent in each sleep stage: Light, Deep, and Rapid-Eye-Movement [REM], with greater time spent in restorative (Deep, REM] stages associated with lower allostatic load index	Yes

## 6 Limitations and knowledge gaps

As the allostatic load model gains traction in military field training research, several limitations and knowledge gaps remain that must be addressed to support its practical application and clarify its potential role as a mechanism for training-related maladaptation. First, it has been observed that the ALI remains underused in military personnel during training courses (Feigel et al., 2025a). Future research measuring ALI and determining its role on psycho-physical outcomes is warranted. Additionally, future research assessing the linkage between the ALI and wearable-derived signals is warranted to determine whether commercial wearable signals can suit allostatic load measurement in military settings. Further, whether the ALI is associated with different psycho-physical outcomes between sexes (Feigel et al., 2025a), and whether different wearable signals are associated with outcomes and ALI between sexes remains unknown. However, future research on determining which wearable may consider the recommendations provided on Table 5, Friedl (2018), or adopt similar valid wrist-worn models adopted from Feigel et al. (2024a) for “wear and forget” interventions.

Second, wrist-worn commercial wearables produce *in vivo* data that are temporal and dynamic wherein averages of time-series data may neglect the dynamic characteristics of physiological time-series data (Feigel et al., 2024a). These characteristics include linear serial dependencies such as trends, rhythms, and autoregressive dynamics. Non-linear characteristics (Richman and Moorman, 2000), such as changes in complexity (Young and Benton, 2015), are inherent to wearable device data (Gronwald et al., 2021), which can indicate critical increases or decreases in fluctuation (i.e., heart rate) (Gronwald et al., 2020) and can therefore be useful in revealing the most stressful impacts that stretch allostasis and result in allostatic load. Consideration should be given to how dynamic instruments can provide data to improve our understanding of allostasis and allostatic load. Understanding how allostasis works in everyday life from a dynamic standpoint can help to prevent allostatic load and its consequences.

Third, allostasis and allostatic load research is dominated by population-based designs, such as cross-sectional, case-control or longitudinal studies (Juster et al., 2010; Carbone et al., 2022). Intense within-individual analysis applying time series analysis is very rare and may reveal when and under which circumstances allostatic loading occurs (i.e., reduced physical performance with blunted heart rate variability and high ALI).

Fourth, allostatic load may not be the only model to adopt in explaining military training-related maladaptation (Selye, 1950; Bates et al., 2013). Previous frameworks have been used in military health sectors to bring actionable and measurable risk factors to the forefront of prevention (Bates et al., 2013; Deuster and OʼConnor, 2015; Givens et al., 2023b; Jonas et al., 2010). However, such frameworks may not contain biological variables that can quantitatively link a unified mechanism with psycho-physiological outcomes. For example, Bates et al. (2013) introduced the Military Demand-Resource Model (MDR) which, using the Conservation of Resources Theory and the Job Demand Resource Model, was developed for military personnel outside combat roles or for trainees. This model conceptualized how demands, such as information overload, non-combat tasks, and resources (i.e., external: leadership, training; internal: awareness, coping, engagement) interact to affect psychological wellbeing, resilience, and cognitive performance. Although the model addresses psychological aspects and operator performance, it does not consider musculoskeletal health or physical fitness (Bates et al., 2013). Hence, the adoption of allostatic load may be able to provide quantifiable means (i.e., ALI, wearable-assessed signals) to link stress with important military health outcomes for job-role performance. However, the allostatic load model may not be able to explain other non-stress-related military training health outcomes, such as acute traumatic MSKIs from a fall or trauma (Jones et al., 2010). Nevertheless, the allostatic load model may outline a promising biologically-grounded mechanism to explain stress-related health outcomes for future study.

Additionally, Schulkin (2004a) purports that the allostasis and allostatic load models are more advantageous than the more common stress regulation model, homeostasis (Schulkin, 2004a). Homeostasis and allostasis aim to achieve physiologic stability (Schulkin, 2004b). However, each model employs different methods to meet this objective that may render allostasis and allostatic load more favorable for adoption in explaining military training-related maladaptation. Homeostasis leverages negative feedback mechanisms to render setpoints stable (static) and return deviations to pre-stressor and normal levels (Schulkin, 2004a). In contrast, allostasis exemplifies the principle of maintaining stability through constant variation (dynamic) of all the parameters of its internal milieu and appropriately matching them to environmental demands (“stability through change”) to allow for subsequent adaptation (Figure 1). Hence, the allostasis model emphasizes a dynamic rather than static principle to achieve biological setpoints and considers the brain a central component in feedback regulation, for whole-body adaptation to contexts (Schulkin, 2004b). Indeed, the brain plays a central role in allostasis (McEwen and Gianaros, 2011; Cohen et al., 2016; Korte et al., 2005). The brain controls the mechanisms across systems via the activity of mediators of allostasis to induce constant variation of parameters in response to stress (McEwen, 2003). Allostasis enforces that the brain designates command by modulating the extent of allostasis via influential factors (experience, memories), individualization (perception of stress, physical condition of the body) (McEwen B. S., 1998), and re-evaluation of needs by anticipating physiological requirements before the behavior (Drug Addiction, 2025). In contrast, the homeostasis model views each organ system as independent from the brain without any influence of modulation (Schulkin, 2004b).

Together, addressing these gaps and limitations may generate the empirical support needed to advance the allostatic load model to the forefront within military sectors and practice—not only within the US but across international Armed Forces—as an alternative framework for better understanding how military-training-related stress may instigate maladaptive health outcomes. By measuring and testing allostatic load against such outcomes, research physiologists can help develop interventions to mitigate allostatic load and enhance post-training military readiness.

## 7 Conclusion

Research physiologists use theoretical models to test new empirical relationships between physiological variables and psycho-physiological outcomes and compare observed outcomes with theoretical predictions to support or refute models. Allostatic load is a model outlining a biological process whereby physiological stability fails owing to recurrent and chronic stress exposure. This model may be a suitable framework to assess its role on musculoskeletal, physical performance and psychological maladaptation during training. Military-training-related stressors, such as energy restriction, cognitive stress, physical overtraining, and sleep deprivation, disrupts neuroendocrine, immune, and autonomic systems, and contributes to allostatic load as a potential mechanism underlying military training-related maladaptation. However, although epidemiological studies on allostatic load measured by ALI demonstrate relationships with poorer physical performance and psychological and musculoskeletal health, further empirical research in military training populations is warranted to support this model. Owing to the limitations of assessing allostatic load via ALI in occupational settings and longitudinal study designs, future research in military training environments may benefit from wrist-worn, commercial wearable technology as opposed to the ALI to measure downstream cardiometabolic and neurobehavioral responses perturbed by stress systems. However, wearable signals should be verified based on the formal definition of allostatic load by determining whether the signals are associated with the ALI and whether signals are associated with secondary and/or tertiary outcomes. The digital phenotype of allostatic load, characterized by one or more digital signatures, such as chronically elevated and variable cardiometabolic activity and altered neurobehavioral responses, requires further testing before it can be implemented in military training as an “at-risk” indicator. Further research could help military practitioners and leadership identify personnel in need of intervention and combat mitigate allostatic load and prevent maladaptive psycho-physical outcomes.

## Data Availability

The original contributions presented in the study are included in the article/supplementary material, further inquiries can be directed to the corresponding author.

## References

[B1] Abouzeid M. Kelsall H. L. Forbes A. B. Sim M. R. Creamer M. C. (2012). Posttraumatic stress disorder and hypertension in Australian veterans of the 1991 gulf war. J. Psychosomatic Res. 72 (1), 33–38. 10.1016/j.jpsychores.2011.08.002 22200520

[B279] Ackerman K. E. Patel K. T. Guereca G. Pierce L. Herzog D. B. Misra M. (2013). Cortisol secretory parameters in young exercisers in relation to LH secretion and bone parameters. Clin. Endocrinol. (Oxf). 78 (1), 114–119. 22671919 10.1111/j.1365-2265.2012.04458.x PMC3443505

[B2] Adler A. B. Williams J. McGurk D. Moss A. Bliese P. D. (2015). Resilience training with soldiers during basic combat training: randomisation by platoon. Appl. Psychol. Health Well Being 7 (1), 85–107. 10.1111/aphw.12040 25641899

[B3] Agostinelli P. J. Linder B. A. Frick K. A. Sefton J. M. (2022). Anthropometrics impact army combat fitness test performance in reserve officer training corps cadets. Mil. Med., usac202. 10.1093/milmed/usac202 35794778

[B287] Akerstedt T. Palmblad J. de la Torre B. Marana R. Gillberg M. (1980). Adrenocortical and gonadal steroids during sleep deprivation. Sleep 3 (1), 23–30. 6781027 10.1093/sleep/3.1.23

[B4] Åkerstedt T. Torsvall L. Gillberg M. (1982). Sleepiness and shift work: field studies. Sleep 5 (Suppl. l_2), S95–S106. 10.1093/sleep/5.s2.s95 6760335

[B5] Alemany J. A. Nindl B. C. Kellogg M. D. Tharion W. J. Young A. J. Montain S. J. (2008). Effects of dietary protein content on IGF-I, testosterone, and body composition during 8 days of severe energy deficit and arduous physical activity. J. Appl. Physiology 105 (1), 58–64. 10.1152/japplphysiol.00005.2008 18450989

[B6] Allison K. F. Keenan K. A. Wohleber M. F. Perlsweig K. A. Pletcher E. R. Lovalekar M. (2017). Greater ankle strength, anaerobic and aerobic capacity, and agility predict Ground Combat Military Occupational school graduation in female Marines. J. Sci. Med. Sport 20, S85-S90–90. 10.1016/j.jsams.2017.08.005 28899656

[B7] Andrzejak S. E. Lewis-Thames M. W. Langston M. E. Han Y. Khan S. Nettles D. A. (2023). The role of BMI in allostatic load and risk of cancer death. Am. J. Prev. Med. 65 (3), 417–426. 10.1016/j.amepre.2023.03.002 36889531 PMC10440242

[B9] Arhakis A. Karagiannis V. Kalfas S. (2013). Salivary alpha-amylase activity and salivary flow rate in young adults. Open Dent. J. 7, 7–15. 10.2174/1874210601307010007 23524385 PMC3601341

[B10] Armstrong L. E. Bergeron M. F. Lee E. C. Mershon J. E. Armstrong E. M. (2021). Overtraining syndrome as a complex systems phenomenon. Front. Netw. Physiol. 1, 794392. 10.3389/fnetp.2021.794392 36925581 PMC10013019

[B12] Balbo M. Leproult R. Van Cauter E. (2010). Impact of sleep and its disturbances on hypothalamo-pituitary-adrenal axis activity. Int. J. Endocrinol. 2010, 759234. 10.1155/2010/759234 20628523 PMC2902103

[B13] Barrett T. J. Sobhani M. Fox G. R. Files B. Patitsas N. Duhaime J. (2022). Diverse predictors of early attrition in an elite marine training school. Mil. Psychol. 34 (4), 388–397. 10.1080/08995605.2021.1993721 38536294 PMC10013366

[B14] Bartlett J. L. Phillips J. Galarneau M. R. (2015). A descriptive study of the US Marine corps fitness tests (2000–2012). Mil. Med. 180 (5), 513–517. 10.7205/MILMED-D-14-00490 25939104

[B15] Bates M. J. Fallesen J. J. Huey W. S. Packard G. A. Ryan D. M. Burke C. S. (2013). Total force fitness in units part 1: military demand-resource model. Mil. Med. 178 (11), 1164–1182. 10.7205/MILMED-D-12-00519 24183762

[B16] Beckie T. M. Duffy A. Groer M. W. (2016). The relationship between allostatic load and psychosocial characteristics among women veterans. Women’s Health Issues. 26 (5), 555–563. 10.1016/j.whi.2016.05.008 27444339

[B17] Beckner M. E. Lieberman H. R. Hatch-McChesney A. Allen J. T. Niro P. J. Thompson L. A. (2023). Effects of energy balance on cognitive performance, risk-taking, ambulatory vigilance and mood during simulated military sustained operations (SUSOPS). Physiol. Behav. 258, 114010. 10.1016/j.physbeh.2022.114010 36349660

[B18] Bei B. Seeman T. E. Carroll J. E. Wiley J. F. (2017). Sleep and physiological dysregulation: a closer look at sleep intraindividual variability. Sleep 40 (9), zsx109. 10.1093/sleep/zsx109 28651371 PMC5806573

[B19] Ben-Avraham R. Afek A. Berezin Cohen N. Davidov A. Van Vleet T. Jordan J. (2022). Feasibility and preliminary effectiveness of Mobile cognitive control training during basic combat training in the military. Mil. Psychol. 34 (1), 55–67. 10.1080/08995605.2021.1969162 38536343 PMC10013478

[B20] Bender A. M. Lawson D. Werthner P. Samuels C. H. (2018). The clinical validation of the athlete sleep screening questionnaire: an instrument to identify athletes that need further sleep assessment. Sports Med. Open 4 (1), 23. 10.1186/s40798-018-0140-5 29869021 PMC5986689

[B21] Berger M. Juster R. P. Westphal S. Amminger G. P. Bogerts B. Schiltz K. (2018). Allostatic load is associated with psychotic symptoms and decreases with antipsychotic treatment in patients with schizophrenia and first-episode psychosis. Psychoneuroendocrinology 90, 35–42. 10.1016/j.psyneuen.2018.02.001 29427955

[B22] Berger M. Taylor S. Harriss L. Campbell S. Thompson F. Jones S. (2019). Hair cortisol, allostatic load, and depressive symptoms in Australian Aboriginal and torres strait islander people. Stress 22 (3), 312–320. 10.1080/10253890.2019.1572745 30835590

[B23] Billman G. E. (2020). Homeostasis: the underappreciated and far too often ignored central organizing principle of physiology. Front. Physiol. 11, 200. 10.3389/fphys.2020.00200 32210840 PMC7076167

[B285] Blumert P. A. Crum A. J. Ernsting M. Volek J. S. Hollander D. B. Haff E. E. (2007). The acute effects of twenty-four hours of sleep loss on the performance of national-caliber male collegiate weightlifters. J. Strength Cond. Res. 21 (4), 1146–1154. 18076267 10.1519/R-21606.1

[B24] Bobba-Alves N. Juster R. P. Picard M. (2022). The energetic cost of allostasis and allostatic load. Psychoneuroendocrinology 146, 105951. 10.1016/j.psyneuen.2022.105951 36302295 PMC10082134

[B25] Boermans S. M. Kamphuis W. Delahaij R. Korteling J. E. H. Euwema M. C. (2013). Perceived demands during modern military operations. Mil. Med. 178 (7), 722–728. 10.7205/MILMED-D-12-00463 23820344

[B26] Bongers P. M. de Winter C. R. Kompier M. A. Hildebrandt V. H. (1993). Psychosocial factors at work and musculoskeletal disease. Scand. J. Work Environ. Health 19 (5), 297–312. 10.5271/sjweh.1470 8296178

[B27] Booth C. K. Probert B. Forbes-Ewan C. Coad R. A. (2006). Australian army recruits in training display symptoms of overtraining. Mil. Med. 171 (11), 1059–1064. 10.7205/milmed.171.11.1059 17153542

[B28] Bouget M. Rouveix M. Michaux O. Pequignot J. M. Filaire E. (2006). Relationships among training stress, mood and dehydroepiandrosterone sulphate/cortisol ratio in female cyclists. J. Sports Sci. 24 (12), 1297–1302. 10.1080/02640410500497790 17101532

[B29] Brock J. R. Legg S. J. (1997). The effects of 6 weeks training on the physical fitness of female recruits to the British army. Ergonomics 40 (3), 400–411. 10.1080/001401397188233 9118939

[B30] Bruun-Rasmussen N. E. Napolitano G. Bojesen S. E. Ellervik C. Holmager T. L. F. Rasmussen K. (2024). Correlation between allostatic load index and cumulative mortality: a register-based study of Danish municipalities. BMJ Open 14 (2), e075697. 10.1136/bmjopen-2023-075697 38346879 PMC10862330

[B31] Budde H. Pietrassyk-Kendziorra S. Bohm S. Voelcker-Rehage C. (2010). Hormonal responses to physical and cognitive stress in a school setting. Neurosci. Lett. 474 (3), 131–134. 10.1016/j.neulet.2010.03.015 20226843

[B32] Buffenstein R. Karklin A. Driver H. S. (2000). Beneficial physiological and performance responses to a month of restricted energy intake in healthy overweight women. Physiol. Behav. 68 (4), 439–444. 10.1016/s0031-9384(99)00222-x 10713282

[B33] Bulmer S. Drain J. R. Tait J. L. Corrigan S. L. Gastin P. B. Aisbett B. (2022a). Quantification of recruit training demands and subjective wellbeing during basic military training. IJERPH 19 (12), 7360. 10.3390/ijerph19127360 35742608 PMC9223755

[B34] Bulmer S. Corrigan S. L. Drain J. R. Tait J. L. Aisbett B. Roberts S. (2022b). Characterising psycho-physiological responses and relationships during a military field training exercise. Int. J. Environ. Res. Public Health 19 (22), 14767. 10.3390/ijerph192214767 36429484 PMC9690080

[B35] Burley S. D. Drain J. R. Sampson J. A. Groeller H. (2018). Positive, limited and negative responders: the variability in physical fitness adaptation to basic military training. J. Sci. Med. Sport 21 (11), 1168–1172. 10.1016/j.jsams.2018.06.018 30057366

[B36] Burley S. Drain J. R. Sampson J. A. Nindl B. C. Groeller H. (2020). Effect of a novel low volume, high intensity concurrent training regimen on recruit fitness and resilience. J. Sci. Med. Sport 23 (10), 979–984. 10.1016/j.jsams.2020.03.005 32345543

[B37] Cadegiani F. A. Kater C. E. (2017). Hormonal aspects of overtraining syndrome: a systematic review. BMC Sports Sci. Med. Rehabil. 9, 14. 10.1186/s13102-017-0079-8 28785411 PMC5541747

[B38] Carbone J. T. (2021). Allostatic load and mental health: a latent class analysis of physiological dysregulation. Stress 24 (4), 394–403. 10.1080/10253890.2020.1813711 32835575

[B39] Carbone J. T. Clift J. Alexander N. (2022). Measuring allostatic load: approaches and limitations to algorithm creation. J. Psychosomatic Res. 163, 111050. 10.1016/j.jpsychores.2022.111050 36228435

[B40] Caspar E. A. Lo Bue S. Magalhães De Saldanha da Gama P. A. Haggard P. Cleeremans A. (2020). The effect of military training on the sense of agency and outcome processing. Nat. Commun. 11 (1), 4366. 10.1038/s41467-020-18152-x 32868764 PMC7459288

[B41] Chacko T. P. Toole J. T. Richman S. Spink G. L. Reinhard M. J. Brewster R. C. (2022). Mapping the network biology of metabolic response to stress in posttraumatic stress disorder and obesity. Front. Psychol. 13, 941019. 10.3389/fpsyg.2022.941019 35959009 PMC9362840

[B42] Chen L. lan Zhao Y. Ye P. fei Zhang J. Zou J. zhong (2017). Detecting driving stress in physiological signals based on multimodal feature analysis and kernel classifiers. Expert Syst. Appl., 85. 10.1016/j.eswa.2017.01.040

[B43] Chicharro J. L. López-Mojares L. M. Lucía A. Pérez M. Alvarez J. Labanda P. (1998). Overtraining parameters in special military units. Aviat. Space Environ. Med. 69 (6), 562–568. Available online at: https://pubmed.ncbi.nlm.nih.gov/9641402/ . 9641402

[B44] Chiodo S. Tessitore A. Cortis C. Cibelli G. Lupo C. Ammendolia A. (2011). Stress-related hormonal and psychological changes to official youth taekwondo competitions. Scand. J. Med. Sci. Sports 21 (1), 111–119. 10.1111/j.1600-0838.2009.01046.x 20030779

[B45] Cho H. M. Park H. Dong S. Y. Youn I. (2019). Ambulatory and laboratory stress detection based on raw electrocardiogram signals using a convolutional neural network. Sensors (Basel) 19 (20), 4408. 10.3390/s19204408 31614646 PMC6833036

[B46] Christensen D. S. Zachariae R. Amidi A. Wu L. M. (2022). Sleep and allostatic load: a systematic review and meta-analysis. Sleep. Med. Rev. 64, 101650. 10.1016/j.smrv.2022.101650 35704985

[B260] Cisneros Jr. G. (2023). Use of Fitness Wearables to Measure and Promote Readiness. Available online at: https://health.mil/Reference-Center/Reports/2023/07/24/Use-of-Fitness-Wearables-to-Measure-and-Promote-Readiness .

[B47] Coge M. Neiva H. P. Pereira A. Faíl L. Ribeiro B. Esteves D. (2024). Effects of 34 weeks of military service on body composition and physical fitness in military cadets of Angola. J. Funct. Morphol. Kinesiol 9 (3), 111. 10.3390/jfmk9030111 39051272 PMC11270323

[B48] Cohen S. Kamarck T. Mermelstein R. (1983). A global measure of perceived stress. J. Health Soc. Behav. 24 (4), 385–396. 10.2307/2136404 6668417

[B49] Cohen S. Gianaros P. J. Manuck S. B. (2016). A stage model of stress and disease. Perspect. Psychol. Sci. 11 (4), 456–463. 10.1177/1745691616646305 27474134 PMC5647867

[B50] Collins R. McGrath D. Horner K. Eusebi S. Ditroilo M. (2019). Effect of external counterpulsation on exercise recovery in team sport athletes. Int. J. Sports Med. 40 (8), 511–518. 10.1055/a-0923-9144 31288290

[B51] Conkright W. R. O’Leary T. J. Wardle S. L. Greeves J. P. Beckner M. E. Nindl B. C. (2022). Sex differences in the physical performance, physiological, and psycho-cognitive responses to military operational stress. Eur. J. Sport Sci. 22 (1), 99–111. 10.1080/17461391.2021.1916082 33840352

[B52] Connor K. M. Davidson J. R. T. (2003). Development of a new resilience scale: the connor-davidson resilience scale (CD-RISC). Depress Anxiety 18 (2), 76–82. 10.1002/da.10113 12964174

[B53] Cooper D. C. Campbell M. S. Baisley M. Hein C. L. Hoyt T. (2024). Combat and operational stress programs and interventions: a scoping review using a tiered prevention framework. Mil. Psychol. 36 (3), 253–265. 10.1080/08995605.2021.1968289 38661468 PMC11057676

[B54] Corrigan S. L. Roberts S. Warmington S. Drain J. Main L. C. (2021). Monitoring stress and allostatic load in first responders and tactical operators using heart rate variability: a systematic review. BMC Public Health 21 (1), 1701. 10.1186/s12889-021-11595-x 34537038 PMC8449887

[B55] Corrigan S. L. Roberts S. S. H. Warmington S. A. Drain J. R. Tait J. L. Bulmer S. (2023). Overnight heart rate variability responses to military combat engineer training. Appl. Ergon. 107, 103935. 10.1016/j.apergo.2022.103935 36371929

[B56] Culley K. DaLomba E. (2025). Posting holistic health and fitness reels on social media platforms to improve soldier health in the brigade. Mil. Med. 190 (1–2), e456–e461. 10.1093/milmed/usae413 39255236

[B57] da Costa B. R. Vieira E. R. (2010). Risk factors for work-related musculoskeletal disorders: a systematic review of recent longitudinal studies. Am. J. Ind. Med. 53 (3), 285–323. 10.1002/ajim.20750 19753591

[B58] de Vries H. Kamphuis W. van der Schans C. Sanderman R. Oldenhuis H. (2022). Trends in daily heart rate variability fluctuations are associated with longitudinal changes in stress and somatisation in police officers. Healthcare 10 (1), 144. 10.3390/healthcare10010144 35052307 PMC8776208

[B59] Deeney C. O’Sullivan L. (2009). Work related psychosocial risks and musculoskeletal disorders: potential risk factors, causation and evaluation methods. Work 34 (2), 239–248. 10.3233/WOR-2009-0921 20037236

[B60] Degoutte F. Jouanel P. Bègue R. J. Colombier M. Lac G. Pequignot J. M. (2006). Food restriction, performance, biochemical, psychological, and endocrine changes in judo athletes. Int. J. Sports Med. 27 (1), 9–18. 10.1055/s-2005-837505 16388436

[B61] Deuster P. A. OʼConnor F. G. (2015). Human performance optimization: culture change and paradigm shift. J. Strength Cond. Res. 29 (Suppl. 11), S52–S56. 10.1519/JSC.0000000000001118 26506199

[B62] Dijksma I. Bekkers M. Spek B. Lucas C. Stuiver M. (2020). Epidemiology and financial burden of musculoskeletal injuries as the leading health problem in the military. Mil. Med. 185 (3–4), e480–e486. 10.1093/milmed/usz328 31603239

[B63] Drain J. R. Sampson J. A. Billing D. C. Burley S. D. Linnane D. M. Groeller H. (2015). The effectiveness of basic military training to improve functional lifting strength in new recruits. J. Strength Cond. Res. 29 (Suppl. 11), S173–S177. 10.1519/JSC.0000000000001072 26506184

[B64] Drug Addiction (2025). Dysregulation of reward, and allostasis. Neuropsychopharmacology. 10.1016/S0893-133X(00)00195-0 11120394

[B65] Durguerian A. Filaire E. Drogou C. Sauvet F. Bougard C. Chennaoui M. (2018). Hyperactivity of the sympatho-adrenomedullary system without any modification of the hypothalamic-pituitary-adrenal axis after food restriction among high-level weightlifters. J. Strength and Cond. Res. 32 (6), 1643–1655. 10.1519/JSC.0000000000002038 29194183

[B66] D’Alessio L. Korman G. P. Sarudiansky M. Guelman L. R. Scévola L. Pastore A. (2020). Reducing allostatic load in depression and anxiety disorders: physical activity and yoga practice as Add-On therapies. Front. Psychiatry 11, 501. 10.3389/fpsyt.2020.00501 32581876 PMC7287161

[B67] E Silva F. B. Vaisman M. Ponce T. de Barros T. R. E Silva C. B. Salerno V. P. (2022). A systematic review of hormone levels, biomarkers of cellular injury and oxidative stress in multi-stressor military field training exercises. Archives Endocrinol. Metabolism 66 (3), 382–389. 10.20945/2359-3997000000443 35289515 PMC9832854

[B68] East W. Muraca-Grabowski S. McGurk M. DeGroot D. Hauret K. Greer T. (2017). Baseline soldier physical readiness requirements study. J. Sci. Med. Sport 20, S24–S25. 10.1016/j.jsams.2017.09.076

[B69] Eddy M. D. Hasselquist L. Giles G. Hayes J. F. Howe J. Rourke J. (2015). The effects of load carriage and physical fatigue on cognitive performance. PLoS One 10 (7), e0130817. 10.1371/journal.pone.0130817 26154515 PMC4496096

[B70] Edgar D. T. Gill N. D. Beaven C. M. Zaslona J. L. Driller M. W. (2021). Sleep duration and physical performance during a 6-week military training course. J. Sleep. Res. 30 (6), e13393. 10.1111/jsr.13393 34031933

[B71] Edwards W. B. (2018). Modeling overuse injuries in sport as a mechanical fatigue phenomenon. Exerc Sport Sci. Rev. 46 (4), 224–231. 10.1249/JES.0000000000000163 30001271

[B72] Ee J. S. Culp P. A. Bevis Z. J. Dogbey G. Y. Agnello R. N. Chang M. H. (2023). Chronic pain and childhood adversity experiences among US military personnel. Mil. Med. 188 (Suppl. 6), 561–566. 10.1093/milmed/usad244 37948239

[B73] Ellis E. M. Prather A. A. Grenen E. G. Ferrer R. A. (2019). Direct and indirect associations of cognitive reappraisal and suppression with disease biomarkers. Psychol. Health 34 (3), 336–354. 10.1080/08870446.2018.1529313 30614281 PMC6492247

[B74] Erickson M. L. Wang W. Counts J. Redman L. M. Parker D. Huebner J. L. (2022). Field-based assessments of behavioral patterns during shiftwork in police academy trainees using wearable technology. J. Biol. Rhythms 37 (3), 260–271. 10.1177/07487304221087068 35416084 PMC12291159

[B75] Fatouros I. G. Destouni A. Margonis K. Jamurtas A. Z. Vrettou C. Kouretas D. (2006). Cell-free plasma DNA as a novel marker of aseptic inflammation severity related to exercise overtraining. Clin. Chem. 52 (9), 1820–1824. 10.1373/clinchem.2006.070417 16840584

[B76] Feigel E. D. Bird M. B. Koltun K. J. Lovalekar M. Mi Q. Martin B. J. (2023). Association of clinically-measured and dynamic ankle dorsiflexion assessed by markerless motion capture during the drop-jump task on landing biomechanics and risk of ankle injury in military personnel undergoing 10 weeks of physical training. J. Sci. Med. Sport 26, 476–481. 10.1016/j.jsams.2023.07.012 37574406

[B77] Feigel E. D. Bird M. B. Koltun K. J. Lovalekar M. Forse J. N. Gage C. R. (2024a). Allostatic load is associated with overuse musculoskeletal injury during US marine corps officer candidates school. Med. Sci. Sports Exerc 56 (11), 2220–2229. 10.1249/MSS.0000000000003507 38934495

[B78] Feigel E. D. Sterczala A. J. Krajewski K. T. Sekel N. M. Lovalekar M. Peterson P. A. (2024b). Physiological characteristics predictive of passing military physical employment standard tasks for ground close combat occupations in men and women. Eur. J. Sport Sci. 24 (9), 1247–1259. 10.1002/ejsc.12159 38967991 PMC11369343

[B79] Feigel E. D. Koltun K. J. Lovalekar M. Kargl C. K. Bird M. B. Forse J. N. (2025a). Association of allostatic load measured by allostatic load index on physical performance and psychological responses during arduous military training. Physiol. Rep. 13 (6), e70273. 10.14814/phy2.70273 40110958 PMC11923871

[B80] Feigel E. D. McCarthy A. Lovalekar M. Koltun K. J. Bird M. B. Martin B. J. (2025b). Wearable-assessed biomechanical and physiological demands during load carriage and tactical mobility tasks among Male and female military personnel. Med. Sci. Sports Exerc. 10.1249/MSS.0000000000003770 40440510

[B81] Felix A. S. Nolan T. S. Glover L. M. Sims M. Addison D. Smith S. A. (2023). The modifying role of resilience on allostatic load and cardiovascular disease risk in the Jackson heart study. J. Racial Ethn. Health Disparities 10 (5), 2124–2135. 10.1007/s40615-022-01392-6 36136291 PMC10030384

[B82] Fernández-Garcia B. Lucía A. Hoyos J. Chicharro J. L. Rodriguez-Alonso M. Bandrés F. (2002). The response of sexual and stress hormones of Male pro-cyclists during continuous intense competition. Int. J. Sports Med. 23 (8), 555–560. 10.1055/s-2002-35532 12439770

[B83] Finnell J. E. Lombard C. M. Padi A. R. Moffitt C. M. Wilson L. B. Wood C. S. (2017). Physical *versus* psychological social stress in Male rats reveals distinct cardiovascular, inflammatory and behavioral consequences. PLoS One 12 (2), e0172868. 10.1371/journal.pone.0172868 28241050 PMC5328366

[B84] Fitriani C. R. G. S. Matthews R. (2016). Women in ground close combat. RUSI J. 161 (1), 14–24. 10.1080/03071847.2016.1152117

[B85] Flanagan S. C. Kotwal R. S. Forsten R. D. (2012). Preparing soldiers for the stress of combat. J. Spec. Oper. Med. 12 (2), 33–41. 10.55460/RPAT-ESAK 22707023

[B86] Flatt A. Hornikel B. Esco M. R. (2017). Heart rate variability and psychometric responses to overload and tapering in collegiate sprint-swimmers. J. Sci. Med. Sport 20 (6), 606–610. 10.1016/j.jsams.2016.10.017 27890479

[B87] Forse J. N. Koltun K. J. Bird M. B. Lovalekar M. Feigel E. D. Steele E. J. (2024). Low psychological resilience and physical fitness predict attrition from US marine corps officer candidate School training. Mil. Psychol. 21, 1–10. 10.1080/08995605.2024.2403826 39433479 PMC12562803

[B88] Foster C. Rodriguez-Marroyo J. A. Koning J. J. de (2017). Monitoring training loads: the past, the present, and the future. Int. J. Sports Physiology Perform. 12 (s2), S2–S8. 10.1123/IJSPP.2016-0388 28253038

[B89] Friedl K. E. (2018). Military applications of soldier physiological monitoring. J. Sci. Med. Sport 21 (11), 1147–1153. 10.1016/j.jsams.2018.06.004 29960798

[B90] Friedl K. E. Looney D. P. (2023). With life there is motion. Activity biomarkers signal important health and performance outcomes. J. Sci. Med. Sport 26 (Suppl. 1), S3–S8. 10.1016/j.jsams.2023.01.009 36775676

[B91] Friedl K. E. Moore R. J. Hoyt R. W. Marchitelli L. J. Martinez-Lopez L. E. Askew E. W. (2000). Endocrine markers of semistarvation in healthy lean men in a multistressor environment. J. Appl. Physiology 88 (5), 1820–1830. 10.1152/jappl.2000.88.5.1820 10797147

[B92] Fries E. Hesse J. Hellhammer J. Hellhammer D. H. (2005). A new view on hypocortisolism. Psychoneuroendocrinology 30 (10), 1010–1016. 10.1016/j.psyneuen.2005.04.006 15950390

[B93] Fry A. C. Kraemer W. J. Stone M. H. Warren B. J. Kearney J. T. Maresh C. M. (1993). Endocrine and performance responses to high volume training and amino acid supplementation in elite junior weightlifters. Int. J. Sport Nutr. 3 (3), 306–322. 10.1123/ijsn.3.3.306 8220396

[B282] Fry R. W. Grove J. R. Morton A. R. Zeroni P. M. Gaudieri S. Keast D. (1994). Psychological and immunological correlates of acute overtraining. Br. J. Sports Med. 28 (4), 241–246. 7894955 10.1136/bjsm.28.4.241 PMC1332084

[B94] Gallagher S. Barbe M. F. (2022). The impaired healing hypothesis: a mechanism by which psychosocial stress and personal characteristics increase MSD risk? Ergonomics 65 (4), 573–586. 10.1080/00140139.2021.1974103 34463204 PMC9847256

[B95] Germano M. L. Dos Santos Gomes C. de Souza Barbosa J. F. Neto N. J. Pereira D. S. Ahmed T. (2023). Allostatic load and physical performance in older adults: findings from the international mobility in aging study (IMIAS). Arch. Gerontol. Geriatr. 109, 104961. 10.1016/j.archger.2023.104961 36806404

[B96] Geronimus A. T. Hicken M. Keene D. Bound J. (2006). “weathering” and age patterns of allostatic load scores among blacks and whites in the United States. Am. J. Public Health 96 (5), 826–833. 10.2105/AJPH.2004.060749 16380565 PMC1470581

[B97] Gianaros P. J. Sheu L. K. Uyar F. Koushik J. Jennings J. R. Wager T. D. (2017). A brain phenotype for stressor‐evoked blood pressure reactivity. JAHA 6 (9), e006053. 10.1161/JAHA.117.006053 28835356 PMC5634271

[B98] Giessing L. Oudejans R. R. D. Hutter V. Plessner H. Strahler J. Frenkel M. O. (2020). Acute and chronic stress in daily police service: a three-week N-of-1 study. Psychoneuroendocrinology 122, 104865. 10.1016/j.psyneuen.2020.104865 32961407

[B99] Givens A. C. Bernards J. R. Kelly K. R. (2023a). Characterization of female US marine recruits: Workload, caloric expenditure, fitness, injury rates, and menstrual cycle disruption during bootcamp. Nutrients 15 (7), 1639. 10.3390/nu15071639 37049480 PMC10096956

[B100] Givens M. O’Connor F. G. Deuster P. A. (2023b). Total force fitness summit 2021: maximizing the health and well-being of service members and their families. Mil. Med. 188 (Suppl. 5), 4–7. 10.1093/milmed/usac440 37665586

[B101] Goertzel B. N. Pennachin C. de Souza Coelho L. Maloney E. M. Jones J. F. Gurbaxani B. (2006). Allostatic load is associated with symptoms in chronic fatigue syndrome patients. Pharmacogenomics 7 (3), 485–494. 10.2217/14622416.7.3.485 16610958

[B102] Goldstein D. S. Kopin I. J. (2007). Evolution of concepts of stress. Stress 10 (2), 109–120. 10.1080/10253890701288935 17514579

[B103] Good C. H. Brager A. J. Capaldi V. F. Mysliwiec V. (2020). Sleep in the United States military. Neuropsychopharmacol 45 (1), 176–191. 10.1038/s41386-019-0431-7 31185484 PMC6879759

[B104] Gou Y. Cheng S. Kang M. Zhou R. Liu C. Hui J. (2025). Association of allostatic load with depression, anxiety, and suicide: a prospective cohort study. Biol. Psychiatry 97 (8), 786–793. 10.1016/j.biopsych.2024.09.026 39395472

[B105] Gouin J. P. Glaser R. Malarkey W. B. Beversdorf D. Kiecolt-Glaser J. (2012). Chronic stress, daily stressors, and circulating inflammatory markers. Health Psychol. 31 (2), 264–268. 10.1037/a0025536 21928900 PMC3253267

[B106] Gronwald T. Rogers B. Hoos O. (2020). Fractal correlation properties of heart rate variability: a new biomarker for intensity distribution in endurance exercise and training prescription? Front. Physiol. 11, 550572. 10.3389/fphys.2020.550572 33071812 PMC7531235

[B107] Gronwald T. Berk S. Altini M. Mourot L. Hoos O. Rogers B. (2021). Real-time estimation of aerobic threshold and exercise intensity distribution using fractal correlation properties of heart rate variability: a single-case field application in a former olympic triathlete. Front. Sports Act. Living 3, 668812. 10.3389/fspor.2021.668812 34124661 PMC8193503

[B108] Guidi J. Lucente M. Sonino N. Fava G. A. (2020). Allostatic load and its impact on health: a systematic review. Psychotherapy Psychosomatics 90 (1), 11–27. 10.1159/000510696 32799204

[B109] Guo R. Sun M. Zhang C. Fan Z. Liu Z. Tao H. (2021). The role of military training in improving psychological resilience and reducing depression among college freshmen. Front. Psychiatry 12, 641396. 10.3389/fpsyt.2021.641396 34079481 PMC8166047

[B110] Guyon A. Balbo M. Morselli L. L. Tasali E. Leproult R. L’Hermite-Balériaux M. (2014). Adverse effects of two nights of sleep restriction on the hypothalamic-pituitary-adrenal axis in healthy men. J. Clin. Endocrinol. Metabolism 99 (8), 2861–2868. 10.1210/jc.2013-4254 24823456 PMC4121029

[B111] Habersaat S. Abdellaoui S. Geiger A. M. Urben S. Wolf J. M. (2018). Low subjective social status in the police is linked to health-relevant changes in diurnal salivary alpha-amylase activity in Swiss police officers. Stress 21 (1), 11–18. 10.1080/10253890.2017.1389882 29037115

[B112] Häkkinen K. Alén M. Komi P. V. (1985). Changes in isometric force- and relaxation-time, electromyographic and muscle fibre characteristics of human skeletal muscle during strength training and detraining. Acta Physiol. Scand. 125 (4), 573–585. 10.1111/j.1748-1716.1985.tb07760.x 4091001

[B284] Halson S. L. Lancaster G. I. Jeukendrup A. E. Gleeson M. (2003). Immunological responses to overreaching in cyclists. Med. Sci. Sports Exerc. 35 (5), 854–861. 12750597 10.1249/01.MSS.0000064964.80040.E9

[B113] Hansen Å. M. Andersen L. L. Mendes de Leon C. F. Bruunsgaard H. Lund R. (2016). School education, physical performance in late midlife and allostatic load: a retrospective cohort study. J. Epidemiol. Community Health 70 (8), 748–754. 10.1136/jech-2015-205664 26767409

[B114] Hansen D. A. P. D. PhD S. B. C. Layton M. E. M. D. PhD, Van Dongen, HPA PhD (2021). Sleep deprivation and sleep-onset insomnia are associated with blunted physiological reactivity to stressors. Mil. Med. 186 (Suppl. ment_1), 246–252. 10.1093/milmed/usaa464 33499519 PMC10895409

[B115] Harman E. A. Gutekunst D. J. Frykman P. N. Nindl B. C. Alemany J. A. Mello R. P. (2008). Effects of two different eight-week training programs on military physical performance. J. Strength Cond. Res. 22 (2), 524–534. 10.1519/JSC.0b013e31816347b6 18550970

[B116] Hastings W. J. Almeida D. M. Shalev I. (2022). Conceptual and analytical overlap between allostatic load and systemic biological aging measures: analyses from the national survey of midlife development in the United States. J. Gerontol. A Biol. Sci. Med. Sci. 77 (6), 1179–1188. 10.1093/gerona/glab187 34180993 PMC9159656

[B117] Hauret K. G. Jones B. H. Bullock S. H. Canham-Chervak M. Canada S. (2010). Musculoskeletal injuries description of an under-recognized injury problem among military personnel. Am. J. Prev. Med. 38 (1 Suppl. l), S61–S70. 10.1016/j.amepre.2009.10.021 20117601

[B118] Hauschild V. D. Barnes S. Hauret K. Lee T. Forrest L. Jones B. H. (2018). The etiology of injuries in US army initial entry training. US. Army Med. Dep. J., EBSCOhost. 22. 29. Available online at: https://openurl.ebsco.com/contentitem/gcd:134620414?sid=ebsco:plink:crawler&id=ebsco:gcd:134620414 30623395

[B119] Heilbronn B. Doma K. Sinclair W. Connor J. Irvine-Brown L. Leicht A. (2023). Acute fatigue responses to occupational training in military personnel: a systematic review and meta-analysis. Mil. Med. 188 (5–6), 969–977. 10.1093/milmed/usac144 35639912 PMC10187475

[B120] Hennigar S. R. McClung J. P. Hatch-McChesney A. Allen J. T. Wilson M. A. Carrigan C. T. (2021). Energy deficit increases hepcidin and exacerbates declines in dietary iron absorption following strenuous physical activity: a randomized-controlled cross-over trial. Am. J. Clin. Nutr. 113 (2), 359–369. 10.1093/ajcn/nqaa289 33184627

[B121] Henning P. C. Scofield D. E. Spiering B. A. Staab J. S. Matheny R. W. Smith M. A. (2014). Recovery of endocrine and inflammatory mediators following an extended energy deficit. J. Clin. Endocrinol. Metab. 99 (3), 956–964. 10.1210/jc.2013-3046 24423293

[B122] Herman J. P. McKlveen J. M. Solomon M. B. Carvalho-Netto E. Myers B. (2012). Neural regulation of the stress response: glucocorticoid feedback mechanisms. Braz J. Med. Biol. Res. 45 (4), 292–298. 10.1590/s0100-879x2012007500041 22450375 PMC3854162

[B123] Hinde K. White G. Armstrong N. (2021). Wearable devices suitable for monitoring twenty four hour heart rate variability in military populations. Sensors (Basel) 21 (4), 1061. 10.3390/s21041061 33557190 PMC7913967

[B124] Hockey G. R. (1997). Compensatory control in the regulation of human performance under stress and high workload; a cognitive-energetical framework. Biol. Psychol. 45 (1–3), 73–93. 10.1016/s0301-0511(96)05223-4 9083645

[B125] Hooper D. R. Kraemer W. J. Saenz C. Schill K. E. Focht B. C. Volek J. S. (2017). The presence of symptoms of testosterone deficiency in the exercise-hypogonadal Male condition and the role of nutrition. Eur. J. Appl. Physiol. 117 (7), 1349–1357. 10.1007/s00421-017-3623-z 28470410

[B126] Huovinen J. Tulppo M. Nissilä J. Linnamo V. Häkkinen K. Kyrolainen H. (2009). Relationship between heart rate variability and the serum testosterone-to-cortisol ratio during military service. Eur. J. Sport Sci. 9 (5), 277–284. 10.1080/17461390902874040

[B127] Huovinen H. T. Hulmi J. J. Isolehto J. Kyröläinen H. Puurtinen R. Karila T. (2015). Body composition and power performance improved after weight reduction in Male athletes without hampering hormonal balance. J. Strength Cond. Res. 29 (1), 29–36. 10.1519/JSC.0000000000000619 25028999

[B128] Hynynen E. Uusitalo A. Konttinen N. Rusko H. (2006). Heart rate variability during night sleep and after awakening in overtrained athletes. Med. and Sci. Sports and Exerc. 38 (2), 313–317. 10.1249/01.mss.0000184631.27641.b5 16531900

[B129] Impellizzeri F. M. Marcora S. M. Coutts A. J. (2019). Internal and external training load: 15 years on. Int. J. Sports Physiol. Perform. 14 (2), 270–273. 10.1123/ijspp.2018-0935 30614348

[B130] Irwin M. R. Olmstead R. Carroll J. E. (2016). Sleep disturbance, sleep duration, and inflammation: a systematic review and meta-analysis of cohort studies and experimental sleep deprivation. Biol. Psychiatry 80 (1), 40–52. 10.1016/j.biopsych.2015.05.014 26140821 PMC4666828

[B131] Ishitobi Y. Akiyoshi J. Tanaka Y. Ando T. Okamoto S. Kanehisa M. (2010). Elevated salivary α-amylase and cortisol levels in unremitted and remitted depressed patients. Int. J. Psychiatry Clin. Pract. 14 (4), 268–273. 10.3109/13651501.2010.500737 24917438

[B132] Ispirlidis I. Fatouros I. G. Jamurtas A. Z. Nikolaidis M. G. Michailidis I. Douroudos I. (2008). Time-course of changes in inflammatory and performance responses following a soccer game. Clin. J. Sport Med. 18 (5), 423–431. 10.1097/JSM.0b013e3181818e0b 18806550

[B133] Jenkins Z. M. Castle D. J. Eikelis N. Phillipou A. Lambert G. W. Lambert E. A. (2022). Autonomic nervous system function in women with anorexia nervosa. Clin. Auton. Res. 32 (1), 29–42. 10.1007/s10286-021-00836-z 34762216

[B134] Jensen A. E. Laird M. Jameson J. T. Kelly K. R. (2019). Prevalence of musculoskeletal injuries sustained during marine corps recruit training. Mil. Med. 184 (Suppl. 1), 511–520. 10.1093/milmed/usy387 30901397

[B135] Johnson T. V. Abbasi A. Master V. A. (2013). Systematic review of the evidence of a relationship between chronic psychosocial stress and C-reactive protein. Mol. Diagn Ther. 17 (3), 147–164. 10.1007/s40291-013-0026-7 23615944

[B136] Jonas W. B. O’Connor F. G. Deuster P. Peck J. Shake C. Frost S. S. (2010). Why total force fitness? Mil. Med. 175 (Suppl. 1), 6–13. 10.7205/milmed-d-10-00280

[B137] Jones B. H. Canham-Chervak M. Canada S. Mitchener T. A. Moore S. (2010). Medical surveillance of injuries in the u.s. military descriptive epidemiology and recommendations for improvement. Am. J. Prev. Med. 38 (1 Suppl. l), S42–S60. 10.1016/j.amepre.2009.10.014 20117600

[B138] Jurvelin H. Tanskanen-Tervo M. Kinnunen H. Santtila M. Kyröläinen H. (2020). Training load and energy expenditure during military basic training period. Med. Sci. Sports Exerc 52 (1), 86–93. 10.1249/MSS.0000000000002092 31343524

[B139] Juster R. P. McEwen B. S. Lupien S. J. (2010). Allostatic load biomarkers of chronic stress and impact on health and cognition. Neurosci. Biobehav Rev. 35 (1), 2–16. 10.1016/j.neubiorev.2009.10.002 19822172

[B140] Juster R. P. Sindi S. Marin M. F. Perna A. Hashemi A. Pruessner J. C. (2011). A clinical allostatic load index is associated with burnout symptoms and hypocortisolemic profiles in healthy workers. Psychoneuroendocrinology 36 (6), 797–805. 10.1016/j.psyneuen.2010.11.001 21129851

[B141] Kargl C. K. Gage C. R. Forse J. N. Koltun K. J. Bird M. B. Lovalekar M. (2024). Inflammatory and oxidant responses to arduous military training: associations with stress, sleep, and performance. Med. Sci. Sports Exerc 56 (12), 2315–2327. 10.1249/MSS.0000000000003525 39160702

[B142] Karin O. Raz M. Tendler A. Bar A. Korem Kohanim Y. Milo T. (2020). A new model for the HPA axis explains dysregulation of stress hormones on the timescale of weeks. Mol. Syst. Biol. 16 (7), e9510. 10.15252/msb.20209510 32672906 PMC7364861

[B143] Karlamangla A. S. Singer B. H. McEwen B. S. Rowe J. W. Seeman T. E. (2002). Allostatic load as a predictor of functional decline. MacArthur studies of successful aging. J. Clin. Epidemiol. 55 (7), 696–710. 10.1016/s0895-4356(02)00399-2 12160918

[B144] Kim D. W. Hassett L. M. Nguy V. Allen N. E. (2019). A comparison of activity monitor data from devices worn on the wrist and the waist in people with parkinson’s disease. Mov. Disord. Clin. Pract. 6 (8), 693–699. 10.1002/mdc3.12850 31745480 PMC6856447

[B145] Klaus K. Doerr J. M. Strahler J. Skoluda N. Linnemann A. Nater U. M. (2019). Poor night’s sleep predicts following day’s salivary alpha-amylase under high but not low stress. Psychoneuroendocrinology 101, 80–86. 10.1016/j.psyneuen.2018.10.030 30428443

[B146] Knapik J. J. Pope R. Orr R. Schram B. (2018). Osteoarthritis: pathophysiology, prevalence, risk factors, and exercise for reducing pain and disability. J. Spec. Oper. Med. 18 (3), 94–102. 10.55460/V9VN-I71T 30222846

[B147] Knauft K. Waldron A. Mathur M. Kalia V. (2021). Perceived chronic stress influences the effect of acute stress on cognitive flexibility. Sci. Rep. 11 (1), 23629. 10.1038/s41598-021-03101-5 34880363 PMC8654917

[B148] Koltun K. J. Sekel N. M. Bird M. B. Lovalekar M. Mi Q. Martin B. J. (2022). Tibial bone geometry is associated with bone stress injury during military training in men and women. Front. Physiol. 13, 803219. 10.3389/fphys.2022.803219 35222074 PMC8874318

[B149] Koltun K. J. Bird M. B. Lovalekar M. Martin B. J. Mi Q. Nindl B. C. (2023). Changes in eating pathology symptoms during initial military training in men and women and associations with BMI and injury risk. Eat. Behav. 48, 101687. 10.1016/j.eatbeh.2022.101687 36463664

[B150] Korte S. M. Koolhaas J. M. Wingfield J. C. McEwen B. S. (2005). The Darwinian concept of stress: benefits of allostasis and costs of allostatic load and the trade-offs in health and disease. Neurosci. and Biobehav. Rev. 29 (1), 3–38. 10.1016/j.neubiorev.2004.08.009 15652252

[B151] Kudielka B. M. Buske-Kirschbaum A. Hellhammer D. H. Kirschbaum C. (2004). HPA axis responses to laboratory psychosocial stress in healthy elderly adults, younger adults, and children: impact of age and gender. Psychoneuroendocrinology 29 (1), 83–98. 10.1016/s0306-4530(02)00146-4 14575731

[B281] Laughlin G. A. Yen S. S. (1996). Nutritional and endocrine-metabolic aberrations in amenorrheic athletes. J. Clin. Endocrinol Metab. 81 (12), 4301–4309. 8954031 10.1210/jcem.81.12.8954031

[B152] Lennartsson A. K. Kushnir M. M. Bergquist J. Jonsdottir I. H. (2012). DHEA and DHEA-S response to acute psychosocial stress in healthy men and women. Biol. Psychol. 90 (2), 143–149. 10.1016/j.biopsycho.2012.03.003 22445967

[B153] Lennartsson A. K. Arvidson E. Börjesson M. Jonsdottir I. H. (2022). DHEA-S production capacity in relation to perceived prolonged stress. Stress 25 (1), 105–112. 10.1080/10253890.2021.2024803 35037820

[B154] Lindsey B. Shaul Y. Martin J. (2025). Salivary biomarkers of tactical athlete readiness: a systematic review. PLoS One 20 (4), e0321223. 10.1371/journal.pone.0321223 40299918 PMC12040155

[B155] Logan J. G. Barksdale D. J. (2008). Allostasis and allostatic load: expanding the discourse on stress and cardiovascular disease. J. Clin. Nurs. 17 (7b), 201–208. 10.1111/j.1365-2702.2008.02347.x 18578796

[B280] Loucks A. B. Verdun M. Heath E. M. (1985). Low energy availability, not stress of exercise, alters LH pulsatility in exercising women. J. Appl. Physiol. 84 (1), 37–46. 9451615 10.1152/jappl.1998.84.1.37

[B156] Lovalekar M. Johnson C. D. Eagle S. Wohleber M. F. Keenan K. A. Beals K. (2018). Epidemiology of musculoskeletal injuries among US air force special tactics operators: an economic cost perspective. BMJ Open Sport Exerc Med. 4 (1), e000471. 10.1136/bmjsem-2018-000471 30622731 PMC6307598

[B157] Lovalekar M. Hauret K. Roy T. Taylor K. Blacker S. D. Newman P. (2021). Musculoskeletal injuries in military personnel-descriptive epidemiology, risk factor identification, and prevention. J. Sci. Med. Sport 24 (10), 963–969. 10.1016/j.jsams.2021.03.016 33824080

[B158] Lovalekar M. Bird M. B. Koltun K. J. (2023). Sex differences in musculoskeletal injury epidemiology and subsequent loss of tactical readiness during marine corps officer candidates school. BMJ Mil. Health 19, e002392. 10.1136/military-2023-002392 37336580

[B159] Magtibay K. Umapathy K. (2023). A review of tools and methods for detection, analysis, and prediction of allostatic load due to workplace stress. IEEE Trans. Affect. Comput., 1–20. 10.1109/TAFFC.2023.3273201

[B160] Main L. C. McLoughlin L. T. Flanagan S. D. Canino M. C. Banks S. (2023). Monitoring cognitive function in the fatigued warfighter: a rapid review of cognitive biomarkers. J. Sci. Med. Sport 26 (Suppl. 1), S54–S63. 10.1016/j.jsams.2023.04.009 37236820

[B161] Maloney E. M. Gurbaxani B. M. Jones J. F. de Souza Coelho L. Pennachin C. Goertzel B. N. (2006). Chronic fatigue syndrome and high allostatic load. Pharmacogenomics 7 (3), 467–473. 10.2217/14622416.7.3.467 16610956

[B162] Man I. S. C. Shao R. Hou W. K. Xin Li S. Liu F. Y. Lee M. (2023). Multi-systemic evaluation of biological and emotional responses to the trier social stress test: a meta-analysis and systematic review. Front. Neuroendocrinol. 68, 101050. 10.1016/j.yfrne.2022.101050 36410619

[B163] Martucci M. Conte M. Ostan R. Chiariello A. Miele F. Franceschi C. (2020). Both objective and paradoxical insomnia elicit a stress response involving mitokine production. Aging (Albany NY) 12 (11), 10497–10505. 10.18632/aging.103274 32420904 PMC7346035

[B164] Mauss D. Jarczok M. N. (2021). The streamlined allostatic load index is associated with perceived stress in life - findings from the MIDUS study. Stress 24 (4), 404–412. 10.1080/10253890.2020.1869935 33504263

[B165] Mažeikienė A. Bekesiene S. Karčiauskaitė D. Mazgelytė E. Larsson G. Petrėnas T. (2021). The association between endogenous hair steroid hormones and social environmental factors in a group of conscripts during basic military training. Int. J. Environ. Res. Public Health 18 (22), 12239. 10.3390/ijerph182212239 34831996 PMC8625620

[B166] Mazurak N. Enck P. Muth E. Teufel M. Zipfel S. (2011). Heart rate variability as a measure of cardiac autonomic function in anorexia nervosa: a review of the literature. Eur. Eat. Disord. Rev. 19 (2), 87–99. 10.1002/erv.1081 25363717

[B167] McEwen S. E. (1993). Stress and the individual. Mechanisms leading to disease. Arch. Intern Med. 153 (18), 2093–2101. 10.1001/archinte.1993.00410180039004 8379800

[B168] McEwen B. S. (1998a). Protective and damaging effects of stress mediators. N. Engl. J. Med. 338 (3), 171–179. 10.1056/NEJM199801153380307 9428819

[B169] McEwen B. S. (1998b). Stress, adaptation, and disease: allostasis and allostatic load. Ann. N. Y. Acad. Sci. 840 (1), 33–44. 10.1111/j.1749-6632.1998.tb09546.x 9629234

[B170] McEwen B. S. (2003). Interacting mediators of allostasis and allostatic load: towards an understanding of resilience in aging. Metabolism 52 (10 Suppl. 2), 10–16. 10.1016/s0026-0495(03)00295-6 14577057

[B171] McEwen B. S. (2006). Sleep deprivation as a neurobiologic and physiologic stressor: allostasis and allostatic load. Metabolism 55 (10 Suppl. 2), S20–S23. 10.1016/j.metabol.2006.07.008 16979422

[B172] McEwen B. (2007). Physiology and neurobiology of stress and adaptation: central role of the brain. Physiol. Rev. 87 (3), 873–904. 10.1152/physrev.00041.2006 17615391

[B173] McEwen G. Gianaros P. J. (2011). Stress- and allostasis-induced brain plasticity. Annu. Rev. Med. 62, 431–445. 10.1146/annurev-med-052209-100430 20707675 PMC4251716

[B174] McFadden B. A. Cintineo H. P. Chandler A. J. Peterson P. Lovalekar M. Nindl B. C. (2024a). United States marine corps recruit training demands associated with performance outcomes. Mil. Med. 189 (Suppl. ment_2), 84–93. 10.1093/milmed/usae124 38920040

[B175] McFadden B. A. Cintineo H. P. Chandler A. J. Mastrofini G. F. Vincenty C. S. Peterson P. (2024b). A sex comparison of the physical and physiological demands of United States marine corps recruit training. Mil. Med. 189 (Suppl. ment_2), 74–83. 10.1093/milmed/usae071 38920031

[B176] McLoughlin S. Kenny R. A. McCrory C. (2020). Does the choice of allostatic load scoring algorithm matter for predicting age-related health outcomes? Psychoneuroendocrinology 120, 104789. 10.1016/j.psyneuen.2020.104789 32739647

[B177] Military Health System (2023). Brandon act. Available online at: https://www.health.mil/Military-Health-Topics/Mental-Health/Brandon-Act.

[B179] Miller G. E. Chen E. Zhou E. S. (2007). If it goes up, must it come Down? Chronic stress and the hypothalamic-pituitary-adrenocortical axis in humans. Psychol. Bull. 133 (1), 25–45. 10.1037/0033-2909.133.1.25 17201569

[B180] Miller R. M. Freitas E. D. S. Heishman A. D. Peak K. M. Buchanan S. R. Bemben D. A. (2022). Associations of serum IL-6 with muscle, bone, and adipose tissue in women. Cytokine 151, 155787. 10.1016/j.cyto.2021.155787 35065509

[B181] Milosevic M. Jovanov E. Frith K. H. (2013). Research methodology for real-time stress assessment of nurses. Comput. Inf. Nurs. 31 (12), 615–621. 10.1097/CIN.0000000000000011 24113163

[B286] Minkel J. Moreta M. Muto J. Htaik O. Jones C. Basner M. (2014). Sleep deprivation potentiates HPA axis stress reactivity in healthy adults. Health Psychol. 33 (11), 1430–1434. 24818608 10.1037/a0034219

[B182] Misra M. Klibanski A. (2014). Endocrine consequences of anorexia nervosa. Lancet Diabetes and Endocrinol. 2 (7), 581–592. 10.1016/S2213-8587(13)70180-3 24731664 PMC4133106

[B183] Misra M. Miller K. K. Kuo K. Griffin K. Stewart V. Hunter E. (2005). Secretory dynamics of ghrelin in adolescent girls with anorexia nervosa and healthy adolescents. Am. J. Physiol. Endocrinol. Metab. 289 (2), E347–E356. 10.1152/ajpendo.00615.2004 15755766

[B184] Molloy J. M. Pendergrass T. L. Lee I. E. Chervak M. C. Hauret K. G. Rhon D. I. (2020a). Musculoskeletal injuries and United States army readiness part I: overview of injuries and their strategic impact. Mil. Med. 185 (9–10), e1461–e1471. 10.1093/milmed/usaa027 32175566

[B185] Molloy J. M. Pendergrass T. L. Lee I. E. Hauret K. G. Chervak M. C. Rhon D. I. (2020b). Musculoskeletal injuries and United States army readiness. Part II: management challenges and risk mitigation initiatives. Mil. Med. 185 (9–10), e1472–e1480. 10.1093/milmed/usaa028 32107561

[B178] Moran J. Smith T. (2019). Military Readiness and Injury Prevention Act of 2019. Available online at: https://www.moran.senate.gov/public/_cache/files/8/2/82ac1b3b-e291-44d2-ae93-875dd5530780/2FC8526981A227A235BD30D2301F1D70.arm19951.pdf .

[B186] Mori T. Karlamangla A. S. Merkin S. S. Crandall C. J. Binkley N. Greendale G. A. (2014). Multisystem dysregulation and bone strength: findings from the study of midlife in the United States. J. Clin. Endocrinol. Metab. 99 (5), 1843–1851. 10.1210/jc.2013-3908 24527715 PMC4010693

[B187] Myllylä M. Parkkola K. I. Ojanen T. Heinonen O. J. Ruohola J. P. Vahlberg T. (2023). Effects of 12-Month training intervention on physical fitness, body composition, and health markers in Finnish navy soldiers. Healthc. (Basel) 11 (19), 2698. 10.3390/healthcare11192698 37830735 PMC10572769

[B188] Nater U. M. Rohleder N. (2009). Salivary alpha-amylase as a non-invasive biomarker for the sympathetic nervous system: current state of research. Psychoneuroendocrinology 34 (4), 486–496. 10.1016/j.psyneuen.2009.01.014 19249160

[B189] Nicolaides N. C. Kyratzi E. Lamprokostopoulou A. Chrousos G. P. Charmandari E. (2015). Stress, the stress system and the role of glucocorticoids. Neuroimmunomodulation 22 (1–2), 6–19. 10.1159/000362736 25227402

[B190] Nindl B. C. Friedl K. E. Frykman P. N. Marchitelli L. J. Shippee R. L. Patton J. F. (1997). Physical performance and metabolic recovery among lean, healthy men following a prolonged energy deficit. Int. J. Sports Med. 18 (5), 317–324. 10.1055/s-2007-972640 9298770

[B191] Nindl B. C. Scofield D. E. Strohbach C. A. Centi A. J. Evans R. K. Yanovich R. (2012). IGF-I, IGFBPs, and inflammatory cytokine responses during gender-integrated Israeli Army basic combat training. J. Strength Cond. Res. 26 (Suppl. 2), S73–S81. 10.1519/JSC.0b013e31825d81ba 22643141

[B192] Nindl B. C. Alvar B. A. Dudley J R. Favre M. W. Martin G. J. Sharp M. A. (2015). Executive summary from the national strength and conditioning association’s second blue ribbon panel on military physical readiness: military physical performance testing. [editorial]. J. Strength and Cond. Res. 10.1519/JSC.0000000000001037 26506191

[B193] Nindl B. C. Beals K. Witchalls J. Friedl K. E. (2017). Military human performance optimization and injury prevention: strategies for the 21st century warfighter. J. Sci. Med. Sport 20 (Suppl. 4), S1-S2–2. 10.1016/j.jsams.2017.10.029 29129335

[B194] Nindl B. C. Billing D. C. Drain J. R. Beckner M. E. Greeves J. Groeller H. (2018). Perspectives on resilience for military readiness and preparedness: report of an international military physiology roundtable. J. Sci. Med. Sport 21 (11), 1116–1124. 10.1016/j.jsams.2018.05.005 29886134

[B195] Oyola M. G. Handa R. J. (2017). Hypothalamic-pituitary-adrenal and hypothalamic-pituitary-gonadal axes: sex differences in regulation of stress responsivity. Stress 20 (5), 476–494. 10.1080/10253890.2017.1369523 28859530 PMC5815295

[B196] O’Connor P. J. Morgan W. P. Raglin J. S. Barksdale C. M. Kalin N. H. (1989). Mood state and salivary cortisol levels following overtraining in female swimmers. Psychoneuroendocrinology 14 (4), 303–310. 10.1016/0306-4530(89)90032-2 2813655

[B197] O’Leary T. J. Saunders S. C. McGuire S. J. Venables M. C. Izard R. M. (2018). Sex differences in training loads during British army basic training. Med. Sci. Sports Exerc 50 (12), 2565–2574. 10.1249/MSS.0000000000001716 30048410

[B198] Pajcin M. Banks S. White J. M. Dorrian J. Paech G. M. Grant C. (2017). Decreased salivary alpha-amylase levels are associated with performance deficits during sleep loss. Psychoneuroendocrinology 78, 131–141. 10.1016/j.psyneuen.2017.01.028 28196342

[B199] Parker H. W. Abreu A. M. Sullivan M. C. Vadiveloo M. K. (2022). Allostatic load and mortality: a systematic review and meta-analysis. Am. J. Prev. Med. 63 (1), 131–140. 10.1016/j.amepre.2022.02.003 35393143

[B200] Pasiakos S. M. Caruso C. M. Kellogg M. D. Kramer F. M. Lieberman H. R. (2011). Appetite and endocrine regulators of energy balance after 2 days of energy restriction: insulin, leptin, ghrelin, and DHEA-S. Obes. (Silver Spring) 19 (6), 1124–1130. 10.1038/oby.2010.316 21212768

[B201] Passi T. Lukander K. Laarni J. Närväinen J. Rissanen J. Vaara J. P. (2022). Effects of overnight military training and acute battle stress on the cognitive performance of soldiers in simulated urban combat. Front. Psychol. 13, 925157. 10.3389/fpsyg.2022.925157 35959037 PMC9360769

[B202] Paxton W. M. M. Nevers J. Nofziger D. Rogers T. Riggs D. S. (2024). Lessons learned from efforts to prevent behavioral health problems and promote mental wellbeing in the US military. Ment. Health and Prev. 34, 200330. 10.1016/j.mhp.2024.200330

[B203] Peake J. M. Kerr G. Sullivan J. P. (2018). A critical review of consumer wearables, Mobile applications, and equipment for providing biofeedback, monitoring stress, and sleep in physically active populations. Front. Physiol. 9, 743. 10.3389/fphys.2018.00743 30002629 PMC6031746

[B204] Piantanida N. A. Knapik J. J. Brannen S. O’Connor F. Injuries during Marine Corps Officer Basic Training (2000). Injuries during marine corps officer basic training. Mil. Med. 165 (7), 515–520. 10.1093/milmed/165.7.515 10920649

[B205] Pihlainen K. Santtila M. Nindl B. C. Raitanen J. Ojanen T. Vaara J. P. (2023). Changes in physical performance, body composition and physical training during military operations: systematic review and meta-analysis. Sci. Rep. 13 (1), 21455. 10.1038/s41598-023-48712-2 38052976 PMC10698179

[B206] Plews D. J. Laursen P. B. Kilding A. E. Buchheit M. (2012). Heart rate variability in elite triathletes, is variation in variability the key to effective training? A case comparison. Eur. J. Appl. Physiol. 112 (11), 3729–3741. 10.1007/s00421-012-2354-4 22367011

[B207] Pope R. P. Herbert R. Kirwan J. D. Graham B. J. (1999). Predicting attrition in basic military training. Mil. Med. 164 (10), 710–714. 10.1093/milmed/164.10.710 10544625

[B208] Pope C. C. Penney D. Smith T. B. (2018). Overtraining and the complexities of coaches’ decision-making: managing elite athletes on the training cusp. Reflective Pract. 19 (2), 145–166. 10.1080/14623943.2017.1361923

[B209] Pritchard J. Després J. P. Gagnon J. Tchernof A. Nadeau A. Tremblay A. (1999). Plasma adrenal, gonadal, and conjugated steroids following long-term exercise-induced negative energy balance in identical twins. Metabolism 48 (9), 1120–1127. 10.1016/s0026-0495(99)90125-7 10484051

[B210] Reichel T. Boßlau T. K. Palmowski J. Eder K. Ringseis R. Mooren F. C. (2020). Reliability and suitability of physiological exercise response and recovery markers. Sci. Rep. 10 (1), 11924. 10.1038/s41598-020-69280-9 32681124 PMC7368084

[B211] Richman J. S. Moorman J. R. (2000). Physiological time-series analysis using approximate entropy and sample entropy. Am. J. Physiol. Heart Circ. Physiol. 278 (6), H2039–H2049. 10.1152/ajpheart.2000.278.6.H2039 10843903

[B212] Roberts A. C. McClure R. D. Weiner R. I. Brooks G. A. (1993). Overtraining affects Male reproductive status. Fertil. Steril. 60 (4), 686–692. 10.1016/s0015-0282(16)56223-2 8405526

[B213] Robinson M. E. Teyhen D. S. Wu S. S. Dugan J. L. Wright A. C. Childs J. D. (2009). Mental health symptoms in combat medic training: a longitudinal examination. Mil. Med. 174 (6), 572–577. 10.7205/milmed-d-02-4108 19585767

[B214] Ross J. M. Watson N. L. Hamlin N. J. Schmidt J. E. (2024). Differences in perceived stress during the COVID-19 pandemic among military dental postgraduate residents. Mil. Med. 189 (11–12), e2700–e2709. 10.1093/milmed/usae270 38776155

[B215] Ruffing K. M. Koltun K. J. De Souza M. J. Williams N. I. (2022). Moderate weight loss is associated with reductions in LH pulse frequency and increases in 24-hour cortisol with no change in perceived stress in young ovulatory women. Physiol. Behav. 254, 113885. 10.1016/j.physbeh.2022.113885 35718216

[B216] Salvador A. Suay F. González-Bono E. Serrano M. A. (2003). Anticipatory cortisol, testosterone and psychological responses to judo competition in young men. Psychoneuroendocrinology 28 (3), 364–375. 10.1016/s0306-4530(02)00028-8 12573302

[B217] Sapolsky R. M. Romero L. M. Munck A. U. (2000). How do glucocorticoids influence stress responses? Integrating permissive, suppressive, stimulatory, and preparative actions. Endocr. Rev. 21 (1), 55–89. 10.1210/edrv.21.1.0389 10696570

[B218] Saxon L. DiPaula B. Fox G. R. Ebert R. Duhaime J. Nocera L. (2020). Continuous measurement of reconnaissance marines in training with custom smartphone app and watch: observational cohort study. JMIR Mhealth Uhealth 8 (6), e14116. 10.2196/14116 32348252 PMC7324996

[B219] Schei E. (1994). A strengthening experience? Mental distress during military service. A study of Norwegian army conscripts. Soc. Psychiatry Psychiatr. Epidemiol. 29 (1), 40–45. 10.1007/BF00796447 8178221

[B220] Schneiderman N. Ironson G. Siegel S. D. (2005). Stress and health: psychological, behavioral, and biological determinants. Annu. Rev. Clin. Psychol. 1, 607–628. 10.1146/annurev.clinpsy.1.102803.144141 17716101 PMC2568977

[B221] Schoofs D. Wolf O. T. (2011). Are salivary gonadal steroid concentrations influenced by acute psychosocial stress? A study using the trier social stress test (TSST). Int. J. Psychophysiol. 80 (1), 36–43. 10.1016/j.ijpsycho.2011.01.008 21256897

[B222] Schorr M. Miller K. K. (2017). The endocrine manifestations of anorexia nervosa: mechanisms and management. Nat. Rev. Endocrinol. 13 (3), 174–186. 10.1038/nrendo.2016.175 27811940 PMC5998335

[B223] Schulkin J. (2004a). Allostasis, homeostasis, and the costs of physiological adaptation (Cambridge: Cambridge University Press). Available online at: https://www.cambridge.org/core/books/allostasis-homeostasis-and-the-costs-of-physiological-adaptation/2E4F34B15CAF45429EFFF1EB57B954FC.

[B224] Schulkin J. (2004b). Allostasis, homeostasis and the costs of physiological adaptation. First paperback edition. Cambridge: University Press.

[B225] Schwarz J. Gerhardsson A. van Leeuwen W. Lekander M. Ericson M. Fischer H. (2018). Does sleep deprivation increase the vulnerability to acute psychosocial stress in young and older adults? Psychoneuroendocrinology 96, 155–165. 10.1016/j.psyneuen.2018.06.003 29982098

[B226] Seeman T. E. Singer B. H. Rowe J. W. Horwitz R. I. McEwen B. S. (1997). Price of adaptation—allostatic load and its health consequences: Macarthur studies of successful aging. Archives Intern. Med. 157 (19), 2259–2268. 10.1001/archinte.1997.00440400111013 9343003

[B227] Seeman T. E. McEwen B. S. Rowe J. W. Singer B. H. (2001). Allostatic load as a marker of cumulative biological risk: Macarthur studies of successful aging. Proc. Natl. Acad. Sci. U. S. A. 98 (8), 4770–4775. 10.1073/pnas.081072698 11287659 PMC31909

[B228] Selye H. (1950). Stress and the general adaptation syndrome. Br. Med. J. 1 (4667), 1383–1392. 10.1136/bmj.1.4667.1383 15426759 PMC2038162

[B229] Selye H. (1976). The stress of life. New York: McGraw-Hill.

[B230] Sharma Ghimire P. Eckart A. Al-Makhzoomy I. K. Stavitz J. (2023). Sex differences in bone, muscle, and inflammatory markers and their associations with muscle performance variables. Sports (Basel) 11 (11), 215. 10.3390/sports11110215 37999432 PMC10675833

[B231] Sher L. D. Geddie H. Olivier L. Cairns M. Truter N. Beselaar L. (2020). Chronic stress and endothelial dysfunction: mechanisms, experimental challenges, and the way ahead. Am. J. Physiology-Heart Circulatory Physiology 319 (2), H488-H506–506. 10.1152/ajpheart.00244.2020 32618516

[B232] Showery J. E. Kusnezov N. A. Dunn J. C. Bader J. O. Belmont P. J. Waterman B. R. (2016). The rising incidence of degenerative and posttraumatic osteoarthritis of the knee in the United States military. J. Arthroplasty 31 (10), 2108–2114. 10.1016/j.arth.2016.03.026 27181491

[B233] Slivka D. R. Hailes W. S. Cuddy J. S. Ruby B. C. (2010). Effects of 21 days of intensified training on markers of overtraining. J. Strength Cond. Res. 24 (10), 2604–2612. 10.1519/JSC.0b013e3181e8a4eb 20733522

[B234] Solianik R. Sujeta A. (2018). Two-day fasting evokes stress, but does not affect mood, brain activity, cognitive, psychomotor, and motor performance in overweight women. Behav. Brain Res. 338, 166–172. 10.1016/j.bbr.2017.10.028 29097329

[B235] Songer T. J. LaPorte R. E. (2000). Disabilities due to injury in the military. Am. J. Prev. Med. 18 (3 Suppl. l), 33–40. 10.1016/s0749-3797(00)00107-0 10736539

[B236] Sproston N. R. Ashworth J. J. (2018). Role of C-Reactive protein at sites of inflammation and infection. Front. Immunol. 9, 754. 10.3389/fimmu.2018.00754 29706967 PMC5908901

[B237] Stephens M. A. C. Wand G. (2012). Stress and the HPA axis: role of glucocorticoids in alcohol dependence. Alcohol Res. 34 (4), 468–483. 23584113 10.35946/arcr.v34.4.11 PMC3860380

[B238] Sterczala A. J. Krajewski K. T. Peterson P. A. Sekel N. M. Lovalekar M. Wardle S. L. (2023). Twelve weeks of concurrent resistance and interval training improves military occupational task performance in men and women. Eur. J. Sport Sci. 0 (0), 2411–2424. 10.1080/17461391.2023.2239752 37517090

[B239] Sterling P. (1988). Allostasis: a new paradigm to explain arousal pathology. Handb. life stress cognition health. 10.1016/j.physbeh.2011.06.004 21684297

[B240] Suárez V. J. C. Pérez J. J. R. (2013). Psycho-physiological response of soldiers in urban combat. Anales. Psicol. 29 (2), 598–603. 10.6018/analesps.29.2.150691

[B241] Szivak T. K. Kraemer W. J. (2015). Physiological readiness and resilience: pillars of military preparedness. J. Strength Cond. Res. 29 (Suppl. 11), S34–S39. 10.1519/JSC.0000000000001073 26506195

[B242] Szivak T. K. Hooper D. R. Dunn-Lewis C. Comstock B. A. Kupchak B. R. Apicella J. M. (2013a). Adrenal cortical responses to high-intensity, short rest, resistance exercise in men and women. J. Strength Cond. Res. 27 (3), 748–760. 10.1519/JSC.0b013e318259e009 22561973

[B243] Szivak T. Kraemer W. Nindl B. Gotshalk L. Volek J. Gomez A. (2013b). Relationships of physical performance tests to military-relevant tasks in women. U. S. Army Med. Dep. J. 20 (6), 20–26. 24706238

[B244] Szivak T. K. Lee E. C. Saenz C. Flanagan S. D. Focht B. C. Volek J. S. (2018). Adrenal stress and physical performance during military survival training. Aerosp. Med. Hum. Perform. 89 (2), 99–107. 10.3357/AMHP.4831.2018 29463354

[B245] Tait J. L. Drain J. R. Bulmer S. Gastin P. B. Main L. C. (2022). Factors predicting training delays and attrition of recruits during basic military training. Int. J. Environ. Res. Public Health 19 (12), 7271. 10.3390/ijerph19127271 35742522 PMC9223722

[B246] Tanaka Y. Ishitobi Y. Maruyama Y. Kawano A. Ando T. Okamoto S. (2012). Salivary alpha-amylase and cortisol responsiveness following electrical stimulation stress in major depressive disorder patients. Prog. Neuropsychopharmacol. Biol. Psychiatry 36 (2), 220–224. 10.1016/j.pnpbp.2011.10.005 22063648

[B247] Tanaka Y. Maruyama Y. Ishitobi Y. Kawano A. Ando T. Ikeda R. (2013). Salivary alpha-amylase and cortisol responsiveness following electrically stimulated physical stress in bipolar disorder patients. Neuropsychiatr. Dis. Treat. 9, 1899–1905. 10.2147/NDT.S48722 24353422 PMC3862394

[B248] Tanskanen M. M. Kyröläinen H. Uusitalo A. L. Huovinen J. Nissilä J. Kinnunen H. (2011). Serum sex hormone-binding globulin and cortisol concentrations are associated with overreaching during strenuous military training. J. Strength Cond. Res. 25 (3), 787–797. 10.1519/JSC.0b013e3181c1fa5d 20543745

[B249] Taylor M. K. Mujica-Parodi L. R. Padilla G. A. Markham A. E. Potterat E. G. Momen N. (2009). Behavioral predictors of acute stress symptoms during intense military training. J. Trauma. Stress 22 (3), 212–217. 10.1002/jts.20413 19479980

[B250] Taylor M. K. Padilla G. A. Hernández L. M. (2017). Anabolic hormone profiles in elite military men: robust associations with age, stress, and fatigue. Steroids 124, 18–22. 10.1016/j.steroids.2017.05.010 28539251

[B251] Taylor D. J. Straud C. Hale W. Grieser E. Garb L. Garb H. (2020). Sleep difficulties as a predictor of attrition in United States air force recruits. Sleep. Health 6 (3), 338–343. 10.1016/j.sleh.2020.01.007 32273194

[B252] Teixeira R. R. Díaz M. M. Santos T. V. da S. Bernardes J. T. M. Peixoto L. G. Bocanegra O. L. (2015). Chronic stress induces a hyporeactivity of the autonomic nervous system in response to acute mental stressor and impairs cognitive performance in business executives. PLoS One 10 (3), e0119025. 10.1371/journal.pone.0119025 25807003 PMC4373764

[B253] Thieux M. Guyon A. Seugnet L. Franco P. (2024). Salivary α-amylase as a marker of sleep disorders: a theoretical review. Sleep. Med. Rev. 74, 101894. 10.1016/j.smrv.2023.101894 38157687

[B283] Tibana R. A. de Almeida L. M. Frade de Sousa N. M. Nascimento D da C. Neto IV de S. de Almeida J. A. (2016). Two consecutive days of crossfit training affects pro and anti-inflammatory cytokines and osteoprotegerin without impairments in muscle power. Front. Physiol. 7, 260. 27445850 10.3389/fphys.2016.00260 PMC4924482

[B254] Tibana R. A. de Sousa N. M. F. Cunha G. V. Prestes J. Fett C. Gabbett T. J. (2018). Validity of session rating perceived exertion method for quantifying internal training load during high-intensity functional training. Sports (Basel) 6 (3), 68. 10.3390/sports6030068 30041435 PMC6162408

[B255] Trevizol A. P. Brietzke E. Grigolon R. B. Subramaniapillai M. McIntyre R. S. Mansur R. B. (2019). Peripheral interleukin-6 levels and working memory in non-obese adults: a post-hoc analysis from the CALERIE study. Nutrition 58, 18–22. 10.1016/j.nut.2018.06.010 30273821

[B11] Tsao T. Hsu C. Yang C. Liou T. (2009). The effect of exercise intensity on serum leptin and c-reactive protein levels. 7 (2), 98–103. Available online at: https://www.webofscience.com/wos/WOSCC/full-record/000272880900004 .

[B256] Tuan T. C. Hsu T. G. Fong M. C. Hsu C. F. Tsai K. K. C. Lee C. Y. (2008). Deleterious effects of short-term, high-intensity exercise on immune function: evidence from leucocyte mitochondrial alterations and apoptosis. Br. J. Sports Med. 42 (1), 11–15. 10.1136/bjsm.2006.029314 17504785

[B257] Turnbull A. V. Rivier C. (1995). Regulation of the HPA axis by cytokines. Brain Behav. Immun. 9 (4), 253–275. 10.1006/brbi.1995.1026 8903845

[B258] Unno K. Tanida N. Ishii N. Yamamoto H. Iguchi K. Hoshino M. (2013). Anti-stress effect of theanine on students during pharmacy practice: positive correlation among salivary α-amylase activity, trait anxiety and subjective stress. Pharmacol. Biochem. Behav. 111, 128–135. 10.1016/j.pbb.2013.09.004 24051231

[B275] U.S. Army Center for Initial Military Training (2023). Holistic Health and Fitness—Soldier Readiness System. Www.Army.Mil. Available online at: https://www.army.mil/article/267256/holistic_health_and_fitness_soldier_readiness_system .

[B259] US Department of Defense (2025). Recruitment rises 12.5% despite ongoing challenges. Available online at: https://www.defense.gov/News/News-Stories/Article/Article/3953052/recruitment-rises-125-despite-ongoing-challenges/https%3A%2F%2Fwww.defense.gov%2FNews%2FNews-Stories%2FArticle%2FArticle%2F3953052%2Frecruitment-rises-125-despite-ongoing-challenges%2F.

[B261] Uusitalo A. L. Uusitalo A. J. Rusko H. K. (1998). Endurance training, overtraining and baroreflex sensitivity in female athletes. Clin. Physiol. 18 (6), 510–520. 10.1046/j.1365-2281.1998.00121.x 9818156

[B262] Vaara J. P. Groeller H. Drain J. Kyröläinen H. Pihlainen K. Ojanen T. (2022). Physical training considerations for optimizing performance in essential military tasks. Eur. J. Sport Sci. 22 (1), 43–57. 10.1080/17461391.2021.1930193 34006204

[B263] van Dalfsen J. H. Markus C. R. (2018). The influence of sleep on human hypothalamic-pituitary-adrenal (HPA) axis reactivity: a systematic review. Sleep. Med. Rev. 39, 187–194. 10.1016/j.smrv.2017.10.002 29126903

[B264] Villanueva A. L. Schlosser C. Hopper B. Liu J. H. Hoffman D. I. Rebar R. W. (1986). Increased cortisol production in women runners. J. Clin. Endocrinol. Metab. 63 (1), 133–136. 10.1210/jcem-63-1-133 3011836

[B265] Vineetha R. Pai K. M. Vengal M. Gopalakrishna K. Narayanakurup D. (2014). Usefulness of salivary alpha amylase as a biomarker of chronic stress and stress related oral mucosal changes - a pilot study. J. Clin. Exp. Dent. 6 (2), e132–e137. 10.4317/jced.51355 24790712 PMC4002342

[B266] Volek J. S. Ratamess N. A. Rubin M. R. Gómez A. L. French D. N. McGuigan M. M. (2004). The effects of creatine supplementation on muscular performance and body composition responses to short-term resistance training overreaching. Eur. J. Appl. Physiol. 91 (5–6), 628–637. 10.1007/s00421-003-1031-z 14685870

[B267] Waldman H. S. Smith J. W. Lamberth J. Fountain B. J. Bloomer R. J. Butawan M. B. (2020). A 28-Day carbohydrate-restricted diet improves markers of cardiovascular disease in professional firefighters. J. Strength Cond. Res. 34 (10), 2785–2792. 10.1519/JSC.0000000000003749 32740289

[B268] Wang X. Yang Q. Liao Q. Li M. Zhang P. Santos H. O. (2020). Effects of intermittent fasting diets on plasma concentrations of inflammatory biomarkers: a systematic review and meta-analysis of randomized controlled trials. Nutrition 79–80, 110974. 10.1016/j.nut.2020.110974 32947129

[B269] Wardle S. L. Greeves J. P. (2017). Mitigating the risk of musculoskeletal injury: a systematic review of the Most effective injury prevention strategies for military personnel. J. Sci. Med. Sport 20 (Suppl. 4), S3-S10–10. 10.1016/j.jsams.2017.09.014 29103913

[B270] Whittle R. S. (2022). Distance travelled by military recruits during basic training is a significant risk factor for lower limb overuse injury. BMJ Mil. Health 168 (5), 343–348. 10.1136/bmjmilitary-2020-001445 32487672

[B271] Wingenfeld K. Schulz M. Damkroeger A. Philippsen C. Rose M. Driessen M. (2010). The diurnal course of salivary alpha-amylase in nurses: an investigation of potential confounders and associations with stress. Biol. Psychol. 85 (1), 179–181. 10.1016/j.biopsycho.2010.04.005 20433894

[B272] Wolf J. M. Nicholls E. Chen E. (2008). Chronic stress, salivary cortisol, and alpha-amylase in children with asthma and healthy children. Biol. Psychol. 78 (1), 20–28. 10.1016/j.biopsycho.2007.12.004 18243483

[B273] Wolpern A. E. Sherwin K. J. Moss W. D. Nygaard I. E. Egger M. J. Brusseau T. A. (2019). Compliance with wrist-worn accelerometers in primiparous early postpartum women. Heliyon 5 (1), e01193. 10.1016/j.heliyon.2019.e01193 30775582 PMC6360339

[B274] Wood E. E. Criss M. M. Byrd-Craven J. (2021). Stress response asymmetries in African American emerging adults exposed to chronic social adversity. Stress 24 (6), 1064–1068. 10.1080/10253890.2021.1955852 34313189 PMC9590254

[B276] Wyss T. Roos L. Hofstetter M. C. Frey F. Ma¨der U. (2014). Impact of training patterns on injury incidences in 12 Swiss army basic military training schools. Mil. Med. 179 (1), 49–55. 10.7205/MILMED-D-13-00289 24402985

[B277] Yasuda N. (2025). Effects of long-term volleyball training on multimodal responses in adolescent female athletes: a follow-up study. J. Sports Med. Phys. Fit. 65 (4), 468–477. 10.23736/S0022-4707.24.16116-6 39787007

[B278] Young H. Benton D. (2015). We should be using nonlinear indices when relating heart-rate dynamics to cognition and mood. Sci. Rep. 5, 16619. 10.1038/srep16619 26565560 PMC4643265

